# Supplementary material for: A nanoscale MOF-based heterogeneous catalytic system for the polymerization of N-carboxyanhydrides enables direct routes toward both polypeptides and related hybrid materials
Source: Nat Commun. 2023 Sep 12;14:5598. doi: 10.1038/s41467-023-41252-3 (PMC10497576; doi:10.1038/s41467-023-41252-3)
Supplement: Supplementary file 1 — Supplementary Information [file 41467_2023_41252_MOESM1_ESM.pdf]

Supplementary Information for

A Nanoscale MOF-Based Heterogeneous Catalytic System for the Polymerization of *N*-Carboxyanhydrides Enables Direct Routes toward Both Polypeptides and Related Hybrid Materials

Ying Liu[1,†], Zhongwu Ren[1,†], Nannan Zhang[1], Xiaoxin Yang[1], Qihua Wu[2], Zehong Cheng[1], Hang Xing[1,*] and Yugang Bai[1,*]

[1] *School of Chemistry and Chemical Engineering, Hunan University, 2 South Lushan Road, Changsha, Hunan 410082, China*

[2] *Jordan Valley Innovation Center, Missouri State University, 524 North Boonville Avenue, Springfield, Missouri 65806, United States*

[†]These authors contributed equally to this work.

Table of Contents

Experimental Procedures ... **2**
- Materials ... 2
- Instrumentation ... 2
- Preparation of MOF nanoparticles ... 2
- Synthesis of NCAs ... 3
- Representative protocol of NCA-ROP with UiO-66 NPs ... 5
- Metal leakage test ... 5
- FT-IR kinetic study of NCA-ROP ... 5
- Preparation of mixed-matrix membranes ... 5
- Mixed-matrix membrane absorption study ... 6
- 4-MUP hydrolysis with UiO-66@PBLG hybrids ... 7
- Copper-catalyzed azide-alkyne cycloaddition (CuAAC) with UiO-67-Cu@PBLG ... 7
- Preparation of MCPA∈ZIF-8@PBLG MMM ... 7
- Sustained release of MCPA from MCPA∈ZIF-8@PBLG MMM ... 7

Supplementary Results and Discussion ... **8**
- Supplementary figures ... 8
- NMR characterizations ... 23
- Additional discussions ... 29

Supplementary References ... **30**

Experimental Procedures

Materials

All the amino acids were purchased from Leyan Store and used as received unless otherwise noted. Triphosgene and acetic acid were purchased from Adamas. Terephthalic acid was purchased from HWRK Chemical. $ZrOCl_2 \cdot 8H_2O$ was purchased from Macklin. All the solvents used in the synthesis were reagent grade and dried with 4Å molecular sieves. Dimethylformamide (DMF) used in the polymerization was reagent grade and dried with 4Å molecular sieves. Dichloromethane (DCM) and chloroform ($CHCl_3$) used in the polymerization were reagent grade, dried with 4Å molecular sieves or used as-is (condition noted in the protocols). Tetrahydrofuran (THF) and hexanes used for NCA recrystallization were purified by distillation. γ-Benzyl-*L*-glutamate *N*-carboxyanhydride (BLG-NCA), γ-benzyl-*D*-glutamate *N*-carboxyanhydride (BDG-NCA), β-benzyl-*L*-aspartic acid *N*-carboxyanhydride (BLA-NCA) and N^{ε}-Cbz-*L*-lysine *N*-carboxyanhydride (ZLL-NCA) were prepared and recrystallized three times following published procedures,[1] but detailed synthetic protocols are also provided in this document for reader's convenience. The prepared NCA monomers were stored at -20°C and used within one month.

Instrumentation

Nuclear magnetic resonance (NMR) spectra were recorded on a Bruker Avance II 400 MHz spectrometer. MestReNova 14 was used to analyze all spectra. Fourier transformation infrared spectroscopy (FT-IR) was performed on an IR Spirit-T spectrometer (Shimadzu). Molecular weight (MW) and dispersity ($Đ$ = M_w/M_n) of polymers were determined by gel permeation chromatography (GPC) equipped with a Waters 515 isocratic pump, three size exclusion columns (PLgel PL1110-6530/6540/6550, Waters), a miniDAWN TREOS II (Wyatt Technology) multi-angle laser light scattering (MALLS) detector, a Waters 2414 refractive index detector, and a Waters 2487 dual-wavelength UV-vis detector. The mobile phase used was DMF containing 0.05 M LiBr at a flow rate of 0.8 mL min^{-1}. The columns were maintained at a temperature of 60 °C. Sample solutions were filtered through a 0.45 μm PTFE filter before analysis. Absolute molecular weights of polymers were determined using ASTRA 7 software (Wyatt Technology). The dn/dc value used for absolute molecular weight calculation of all PBLG or PDLG samples was 0.093, and 0.123 for PZLL samples. All scanning electron microscopy (SEM) images or energy-dispersive X-ray spectroscopy (EDX) analyses were collected from a Zeiss Sigma 300 Scanning Electron Microscope. Matrix-assisted laser desorption-ionization time-of-flight mass spectrometry (MALDI-TOF) was performed on a Bruker UltrafleXtreme Mass Spectrometer (Bruker, Billerica, MA, USA) with 2,5-dihydroxybenzoic acid (DHB) used as the matrix.

Preparation of MOF nanoparticles

UiO-66 nanoparticles. $ZrOCl_2 \cdot 8H_2O$ (161.1 mg, 0.5 mmol) and terephthalic acid (124.6 mg, 0.75 mmol) were dissolved in DMF (30 mL) by ultrasound treatment. Various volumes of acetic acid (7.50 mL, 8.10 mL, 8.50 mL or 9.10 mL) were added as the modulator to adjust the sizes of the obtained UiO-66 nanoparticles, named as UiO-66-1, UiO-66-2, UiO-66-3, UiO-66-4, respectively. The mixed solution was placed in a glass bottle in Teflon-lined autoclave. The reaction autoclave was heated in an oven at 90 °C for 18 h. The product mixture was centrifuged and the obtained UiO-66 nanoparticles were washed with DMF (3 times), acetone (twice), and THF (twice). The obtained UiO-66 nanoparticles further placed in acetone and THF for 24 h to allow solvent exchange. The nanoparticles were finally isolated by centrifugation and dried at 60 °C for 6 h.

Note: the quality of acetic acid significantly affects the size of the resulting NPs.

UiO-67-Cu. 39.1 mg of 2,2-bipyridine-5,5-dicarboxylic acid (H_2BPyDC) and 37.3 mg of $ZrCl_4$ were added into a glass bottle. 40 mL of DMF was then added, and sonication was used to fully disperse the reactants. The glass bottle was placed in the reaction kettle and heated at 100°C for 10 h. The solid product was centrifuged and washed with 80 °C DMF for 5 times, before a solvent exchange to acetonitrile was performed. The product was dried at 60 °C for 6 h to obtain UiO-67(Py). 129.5 mg of UiO-67(Py) and 35.0 mg of CuCl was then added into a glass bottle. 6 mL of acetonitrile was added, and sonication was used to fully disperse the reactants. The glass bottle was placed in the reaction kettle and heated at 80 °C for 72 h. The solid product was centrifuged and washed with 65 °C acetonitrile for 3 times. Finally, the solid was dried at 60 °C for 6 h to obtain UiO-67-Cu.

Other MOFs. Other MOF nanoparticles were synthesized using similar solvothermal reactions. For UiO-67, $ZrOCl_2·8H_2O$ and biphenyl-4,4'-dicarboxylic acid (H_2BPDC) were used, and DMF was used as the solvent. For HKUST-1, $Cu(NO_3)_2·3H_2O$ and trimesic acid (H_3BTC) were used, and a 1:1:1 (v) mixture of DMF-H_2O-EtOH was used as the solvent. For NU-1000, $ZrOCl_2·8H_2O$ and 1,3,6,8-tetrakis(*p*-benzoic acid)pyrene (H_4TBAPy) were used, and DMF was used as the solvent. For MIL-101(Cr), $CrCl_3·6H_2O$ and H_2BDC were used, and H_2O was used as the solvent. For ZIF-8, $Zn(OAc)_2·2H_2O$ and 2-methylimidazole (2-Mim) were used, and H_2O was used as the solvent. All the MOFs are well-established materials with detailed synthetic protocols available in previous literatures.[2-5]

Synthesis of NCAs

Safety statement: triphosgene is highly toxic. Anyone who works with triphosgene should avoid all personal contact including inhalation, and works only in a well-ventilated fume hood. Always wear protective clothing. The container should be securely sealed after use.

All the NCA synthetic protocols are analogous to previously reported protocols in literature.[1] All glasswares used in the synthesis should be thoroughly oven-dried.

Synthesis of *γ*-benzyl glutamate *N*-carboxyanhydrides (BLG-NCA and BDG-NCA)

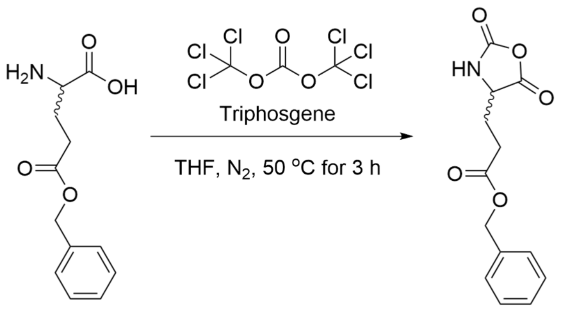

γ-Benzyl-*L*-glutamate or γ-Benzyl-*D*-glutamate (4.74 g, 20.0 mmol) and triphosgene (3.00 g, 10.0 mmol) was added into an oven-dried 100-mL round-bottom flask, into which anhydrous THF (40 mL) was then added under nitrogen protection. The mixture was stirred at 50°C for 3 h under nitrogen. The mixture was cooled down to room temperature, and pure BLG-NCA was obtained by recrystallization in anhydrous THF/hexane for 3 times as a white, needle-like crystalline solid (3.90 g, 74%). The product was stored at -20°C in freezer in a jar filled with desiccants. NMR characterization results for *D*-/*L*-NCAs were identical in achiral solvents. ^1H NMR (400 MHz, $CDCl_3$): δ 7.35 (*m*, 5H), 6.38 (*s*, 1H), 5.15 (*s*, 2H), 4.38 (*m*, 1H), 2.61 (*t*, *J* =

6.4 Hz, 2H), 2.31 (*m*, 1H), 2.14 (*m*, 1H). ^{13}C NMR (101 MHz, $CDCl_3$): δ 172.40, 169.06, 152.03, 135.19, 128.73, 128.61, 127.94, 67.12, 56.82, 29.66, 27.04. High resolution MS (EI, m/z): Calculated for $C_{13}H_{14}NO_5$ ($[M+H]^+$): 264.0872, found: 264.0874.

Synthesis of *N*$^{\varepsilon}$-Cbz-*L*-lysine *N*-carboxyanhydride (ZLL-NCA)

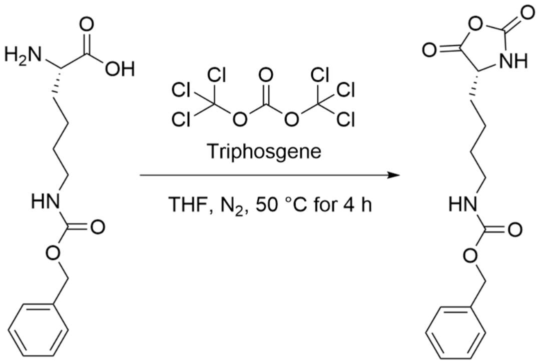

N$^{\varepsilon}$-Cbz-*L*-lysine (2.80 g, 10.0 mmol) and triphosgene (1.50 g, 5.00 mmol) were added into an oven-dried 100-mL round-bottom flask, into which anhydrous THF (30 mL) was added under nitrogen protection. The mixture was stirred at 50°C for 4 h under nitrogen. The mixture was cooled down to room temperature, and pure ZLL-NCA was obtain by recrystallization in anhydrous THF/hexane for 4 times as a white fluffy solid (1.50 g, 50%). The product was stored at -20°C in freezer in a jar filled with desiccants. ^{1}H NMR (400 MHz, DMSO-*d*$_6$): δ 9.08 (*s*, 1H), 7.31 (*m*, 5H), 7.24 (*m*, 1H), 5.01 (*s*, 2H), 4.42 (*m*, 1H), 2.97 (*m*, 2H), 1.68 (*m*, 2H), 1.25-1.45 (*m*, 4H). ^{13}C NMR (101 MHz, $CDCl_3$): δ 170.17, 156.93, 152.56, 136.64, 128.61, 128.25, 128.03, 66.75, 57.48, 40.18, 30.63, 29.14, 21.26. High resolution MS (EI, m/z): Calculated for $C_{15}H_{19}N_2O_5$ ($[M+H]^+$): 307.1294, found: 307.1299.

Synthesis of *β*-benzyl aspartate *N*-carboxyanhydrides (BLA-NCA)

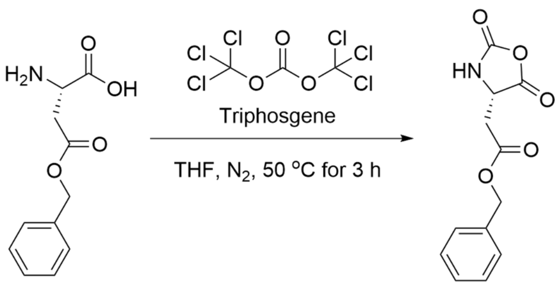

L-aspartic acid *β*-benzyl ester (2.23 g, 10.0 mmol) and triphosgene (1.50 g, 5.00 mmol) were added into an oven-dried 100-mL round-bottom flask, into which anhydrous THF (30 mL) was added under nitrogen protection. The mixture was stirred at 50°C for 4 h under nitrogen. The mixture was cooled down to room temperature, and pure BLA-NCA was obtain by recrystallization in anhydrous THF/hexane for 3 times as a white needle-like crystalline (1.80 g, 67%). The product was stored at -20°C in freezer in a jar filled with desiccants. ^{1}H NMR (400 MHz, $CDCl_3$): δ 7.38 (*m*, 5H), 6.20 (*s*, 1H), 5.19 (*s*, 2H), 4.60 (*m*, 1H), 3.07 (*m*, 1H), 2.85 (*m*, 1H). ^{13}C NMR (101 MHz, $CDCl_3$): δ 169.88, 168.63, 152.02, 135.22, 128.57, 128.50, 128.40, 67.19, 53.60, 35.50. High resolution MS (EI, m/z): Calculated for $C_{12}H_{12}NO_5$ ($[M+H]^+$): 250.0710, found: 250.0708.

Representative protocol of NCA-ROP with UiO-66 NPs

All NCA polymerizations were conducted in a fume hood at room temperature. UiO-66 nanoparticles (3.0 to 30.0 mg, based on the desired mass ratio of BLG-NCA to UiO-66) were pre-dispersed in DCM (500 µL) by ultrasonic treatment for 40 min, then BLG-NCA (30.0 mg, 0.114 mmol) dissolved in DCM (500 µL) was mixed with the UiO-66 suspension. After vigorously stirring for 20 h (or shorter, based on the desired M/I ratio and conversion), the reaction mixture was centrifuged at 13523 rcf for 20 min, and the supernatants were added into cold diethyl ether to precipitate the polypeptide product. After centrifugation and drying under vacuum, the obtained PBLG were analyzed by GPC to determine the M_n and dispersity.

Note: stirring may significantly affect the heterogeneously catalyzed polymerization. For best result, always place the reaction vial/flask in the center of the stirring plate used.

For BLG-NCA polymerizations with $m_{BLG\text{-}NCA}/m_{UiO\text{-}66}$ >10, the mass of UiO-66 nanoparticles used was set at 3 mg, whilst more NCA and solvent were used to keep the concentration of BLG-NCA at 0.114 M. Polymerizations of other NCA monomers or in different solvents by the UiO-66 nanoparticles were performed similarly.

Chain-end functionalization: right after the polymerization, excess 4-nitrophenyl-1-pyrenylmethyl ester (approximately 10 equivalents to PBLG) and 2 µL of triethylamine were added to the reaction, and the mixture was stirred for another 24 h to produce UiO-66@PBLG-Pyrene. The PBLG-Pyrene was isolated and purified by centrifugation and precipitating in MeOH for 3 times.

UiO-66 NPs recycling: after polymerization, the UiO-66 NPs were collected by centrifugation at 13523 rcf for 20 min, then DMF was used as solvent to wash the UiO-66 NPs for another twice. The collected UiO-66 NPs were regenerated by placing in boiling water for 20 min. After cooled down and centrifugation, solvent exchange was by placing the UiO-66 NPs in acetone for 8 h. The UiO-66 NPs were then washed with acetone for 3 times and dried at 50°C in a vacuum oven before used in another polymerization with the same m_{NCA}/m_{MOF} ratio.

Metal leakage test

The extent of metal (Zr) leaching of UiO-66 in DCM or DCM/water mixture was evaluated with the following protocol. 30 mg of UiO-66 nanoparticles was placed in 1 mL of anhydrous DCM, or 1 mL of 10% H_2O/DCM mixture, and the mixtures were ultrasonicated for 1 h followed by vigorous stirring for another 24 h. After centrifugating at 12000 rpm for 30 min, the collected the supernatants were evaporated and prepared for ICP-MS analysis to detect the amount of leaked Zr species. The results of this experiment are listed in Table S1.

FT-IR kinetic study of NCA-ROP

10 µL of the NCA-ROP reaction mixture was extracted from the polymerization vial using a micropipette, and placed onto the KBr window plates for FT-IR spectroscopic measurements. The measurements were repeated at the set time points with certain intervals as desired. Since the IR absorption peak of C=O on the side chain of BLG-NCA was constant during the polymerization, its peak was used as the internal standard. The real-time conversion of BLG-NCA and the yield of PBLG in the reaction were determined by monitoring the absorbance of the characteristic peaks of BLG-NCA at 1784 cm^{-1} and PBLG at 1652 cm^{-1}. All IR kinetic studies were repeated 2 more times.

Preparation of mixed-matrix membranes

To prepare the membrane, a 24 mg/mL PBLG stock solution (39 kDa) in $CHCl_3$ was firstly prepared, and the following sample mixtures were then prepared:

PBLG: The PBLG stock solution (24 mg/mL in $CHCl_3$, 2 mL) was diluted by 2mL of $CHCl_3$ The mixture was mixed thoroughly. The final solution contained 12 mg/mL of PBLG.

UiO-66-Only: UiO-66 nanoparticle (60 mg) was added into $CHCl_3$ (4 mL). The mixture was mixed thoroughly after 4 mL of $CHCl_3$ was added. The final mixture contained 7.5 mg/mL of UiO-66.

UiO-66+PBLG: UiO-66 nanoparticle (60 mg) was added into PBLG stock solution (24 mg/mL in $CHCl_3$, 4 mL). The mixture was mixed thoroughly after 4 mL of $CHCl_3$ was added. The final mixture contained 7.5 mg/mL of UiO-66 and 12 mg/mL of extra-added free standing PBLG.

UiO-66@PBLG: In 2 mL of $CHCl_3$, BLG-NCA (60 mg) was polymerized with 30 mg of UiO-66 nanoparticle. After full consumption of BLG-NCA, 2 mL of $CHCl_3$ was added. The mixture was mixed thoroughly. The final mixture contained 7.5 mg/mL of UiO-66 and 12 mg/mL of PBLG attached to the MOF. Note: at BLG-NCA:MOF = 2:1 mass ratio, the polymerization yielded PBLG of M_n = 39 kDa. Due to the release of CO_2 in the polymerization, 60 mg of BLG-NCA would yield approximately 24 mg of PBLG.

To prepare the membranes, a Nylon-6 substrate membrane (0.2 μm, 25 mm) was placed on a vacuum filtration device (as shown in the picture). Drip one of the above sample mixtures (0.5 mL) evenly onto the substrate membrane. The mixture was gravity-filtered through the membrane until there was no visible $CHCl_3$ on the membrane, then the vacuum was turned on for 1 h to air-dry the membrane. For SEM characterization of the membranes, a 5×5 mm piece of the membrane was cut, sprayed with gold on the surface before visualization under the microscope.

Mixed-matrix membrane absorption study

Methylene blue (MB) aqueous solution (5 mg/L) and $K_2Cr_2O_7$ aqueous solution (10 mg/L) were prepared. The membrane of interest was loaded on a vacuum filtration device (as shown in the figure below), and 10 mL of solution (MB or $K_2Cr_2O_7$) was suction-filtered through the membrane. The filtrate was collected and its residual dye/metal content was quantified by UV-vis spectroscopy, and compared with that of the respective original solution.

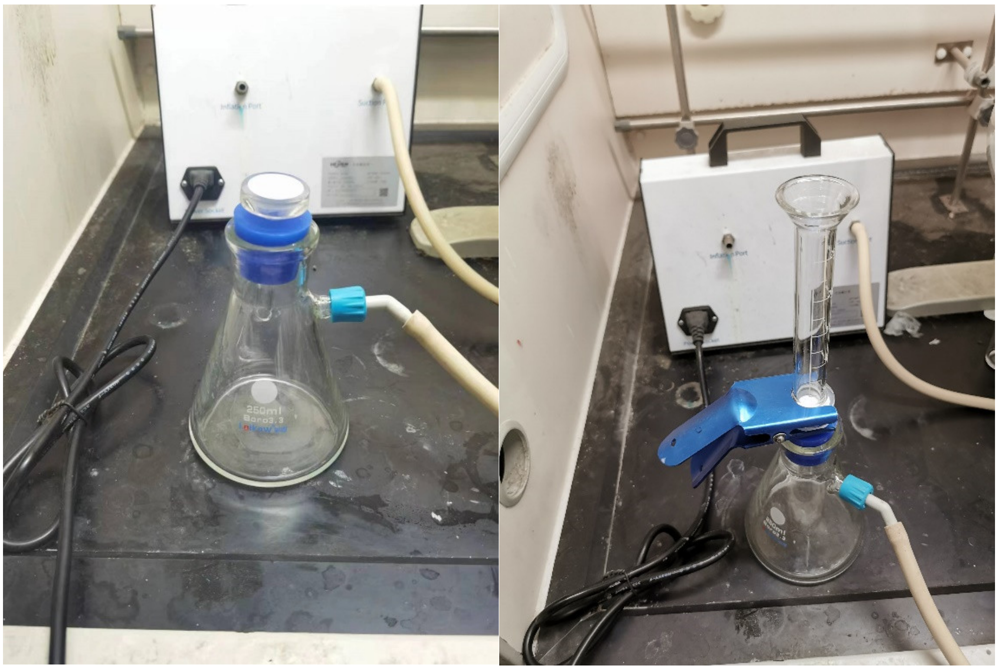

4-MUP hydrolysis with UiO-66@PBLG hybrids

UiO-66@PBLG was firstly synthesized with $m_{BLG\text{-}NCA}/m_{UiO\text{-}66}$ = 30/15 (mg/mg) in 1 mL of DCM by following the polymerization procedures provided above. 4-MUP (8 mg) dissolved in 200 µL of MeOH and 1 mL of 4-methylmorpholine buffer (pH=10) were added into the UiO-66@PBLG solution and stirred for 24 h at room temperature. After the mixture was precipitated in MeOH (4 mL) and centrifugated, the supernatant was collected to detect the hydrolyzed product using a HITACHI F-7000 fluorescence spectrophotometer (E_x: 365 nm, E_m: 450 nm).

Copper-catalyzed azide-alkyne cycloaddition (CuAAC) with UiO-67-Cu@PBLG

UiO-67-Cu@PBLG was synthesized with $m_{BLG\text{-}NCA}/m_{UiO\text{-}67\text{-}Cu}$ = 30/15 (mg/mg) in 1 mL of DCM by following the polymerization procedures provided above. The non-fluorescent 3-AU (20 µM), *p*-ethynylanisole (40 µM) and sodium ascorbate (60 mM) were mixed with 330 µL of the synthesized UiO-67-Cu@PBLG hybrid solution in 700 µL of PBS under N_2 atmosphere. After the reaction was stirred at 40 °C for 24 h, the mixture was centrifuged and the supernatant was collected to detect the fluorescent product using a HITACHI F-7000 fluorescence spectrophotometer (E_x: 410 nm, E_m: 480 nm).

Preparation of MCPA∈ZIF-8@PBLG MMM

MCPA∈ZIF-8. 100 mg of ZIF-8 NPs (300 nm in diameter) and 120 mg of MCPA were mixed in 10 mL of acetone, and the mixture was stirred at room temperature for 24 h. Free MCPA was removed by centrifugation and washing with acetone for 3 times, and the desired product, MCPA∈ZIF-8, was obtained after drying at 50 °C for 4 h.

MCPA∈ZIF-8@PBLG MMM. The MCPA∈ZIF-8@PBLG hybrid was synthesized by using MCPA∈ZIF-8 as the catalyst for the ROP of BLG-NCA. Polymerization with performed using the above-mentioned protocol at $m_{BLG\text{-}NCA}/m_{MCPA\in ZIF\text{-}8}$ = 30/30 (mg/mg) in 1 mL of DCM. After the polymerization was completed, the hybrid solution was air-dried on a 1 × 1.2 cm mold layer by layer to obtain the MCPA∈ZIF-8@PBLG MMM.

Sustained release of MCPA from MCPA∈ZIF-8@PBLG MMM

The release of MCPA was performed by soaking the prepared MCPA∈ZIF-8@PBLG MMM in 5 mL of PBS (pH = 6.0). MCPA∈ZIF-8 was treated in the same way for comparison. For the quantification of released MCPA, the solution was filtered and analyzed with HPLC. HPLC analysis was performed using an Agilent Model 1260 HPLC system with a Phenomenex 5 µm C18 column (2.1 mm × 150 mm held at 40 °C) and the UV signal at 270 nm was used for quantification. The mobile phase including solution A (5 mM aq. ammonium formate) and solution B (5 mM ammonium formate in methanol) was kept at a flow rate of 0.6 mL/min. The HPLC gradient was A/B = 8/2 during 0.0~3.0 min; A/B = 2/8 during 3.1~10 min; A/B = 2/8 during 10~15 min.

Supplementary Results and Discussion

Supplementary figures

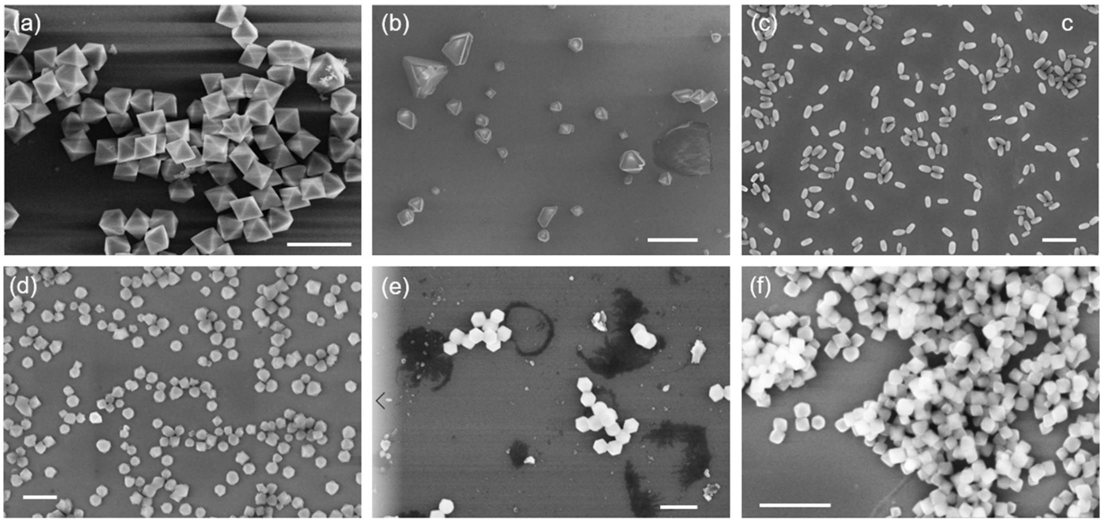

Supplementary Fig. 1. SEM images of as-synthesized (a) UiO-67, (b) HKUST-1, (c) NU-1000, (d) MIL-101(Cr), (e) ZIF-8, and (f) UiO-66. The scale bars represent, from (a) to (e), 5 µm, 25 µm, 1 µm, 500 nm, 1 µm and 500 nm, respectively.

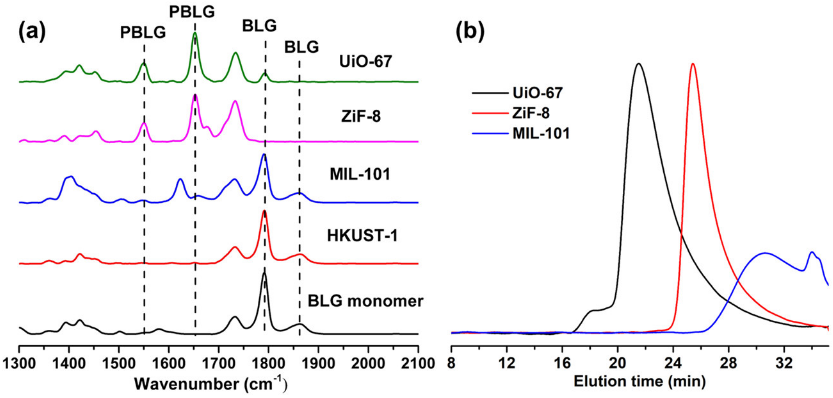

Supplementary Fig. 2. The results of BLG NCA polymerization by various MOF nanoparticles, $m_{BLG\text{-}NCA}/m_{MOF}$ = 5, [BLG-NCA]$_0$ = 0.114 M. (a) FT-IR spectra of the BLG-NCA monomer and the reaction mixtures after NCA-ROP by various MOF nanoparticles for 12 h. (b) GPC curves for the produced polypeptides by UiO-67, ZiF-8 and MIL-101 nanoparticles, respectively.

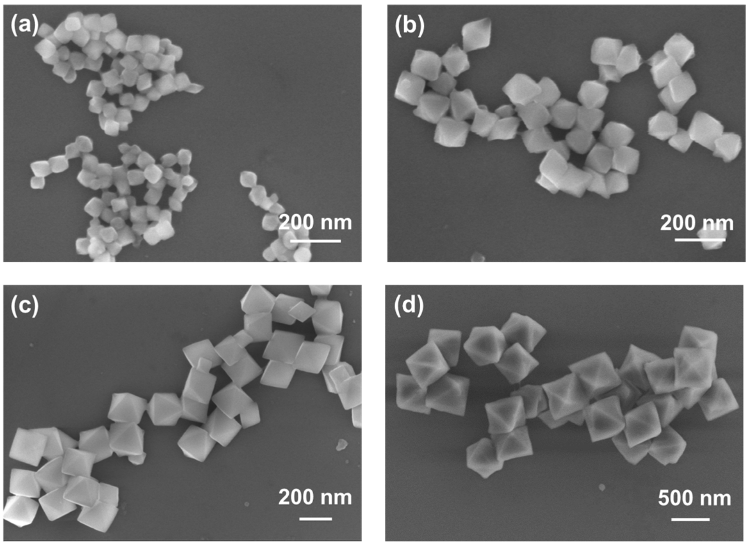

Supplementary Fig. 3. SEM images of as-synthesized UiO-66 nanoparticles of different sizes. (a) UiO-66-1, 60~80 nm. (b) UiO-66-2, 120~150 nm. (c) UiO-66-3, 200~250 nm. (d) UiO-66-4, 550~600 nm.

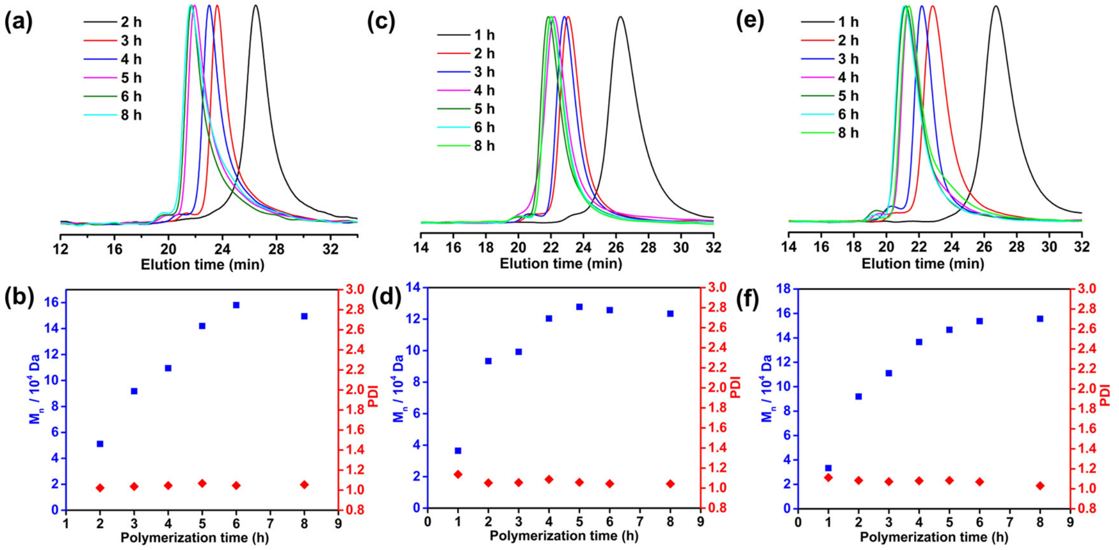

Supplementary Fig. 4. GPC-dRI curves, M_n and dispersity of the PBLGs in the courses of water-initiated NCA-ROPs on UiO-66 nanoparticles. Samples were taken out for GPC characterization every 1 h during the polymerization processes conducted at a fixed $m_{BLG\text{-}NCA}/m_{UiO\text{-}66}$ ratio of 10 in benchtop DCM (not dried). (a-b) Polymerizations done using UiO-66-1, 60~80 nm. (c-d) Polymerizations done using UiO-66-3, 250~300 nm. (e-f) Polymerizations done using UiO-66-4, 550~600 nm.

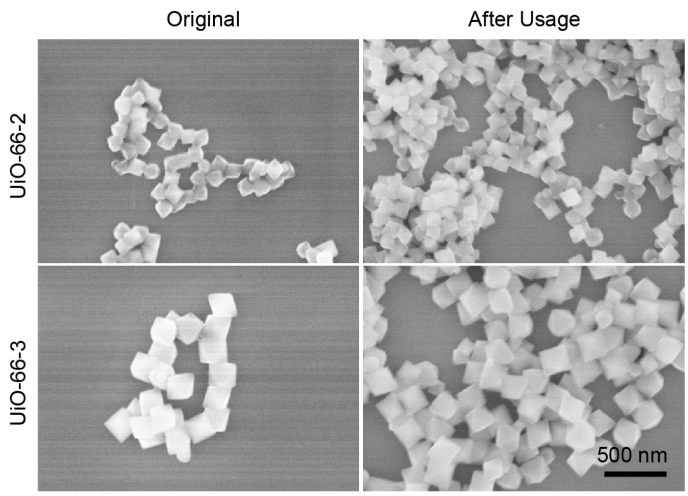

Supplementary Fig. 5. SEM images of UiO-66 before and after being used as the initiation system for NCA polymerization.

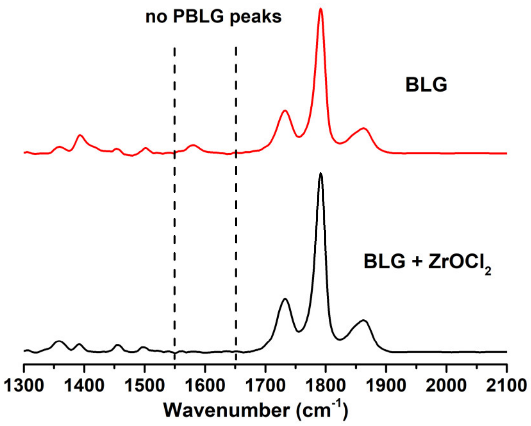

Supplementary Fig. 6. The FT-IR spectra of the BLG-NCA monomer, and the reaction mixture of $ZrOCl_2$ and BLG-NCA after 24 h ($[BLG\text{-}NCA]_0$ = 0.114 M, $m_{BLG\text{-}NCA}/m_{ZrOCl2}$=10).

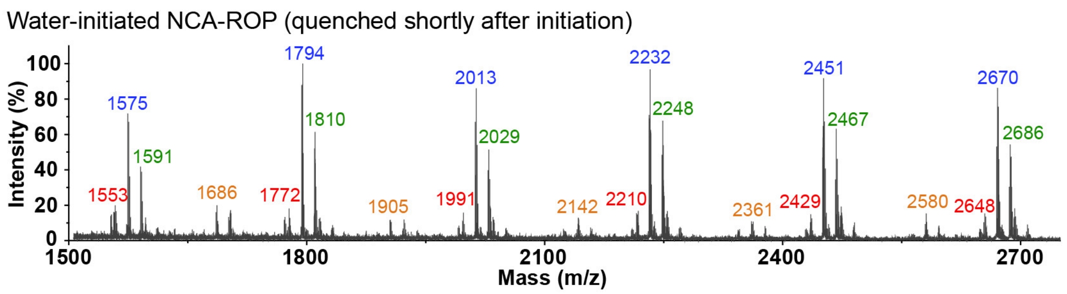

Supplementary Fig. 7. MALDI-TOF MS spectrum of PBDLG with low molecular weight from water-initiated NCA-ROP on UiO-66 NPs. The polymerization was quenched shortly after initiation to control the molecular weight so that MALDI analysis can be facilitated.

Peak assignments (M = H-(NH-($C_{11}H_{12}O_2$)-CO)$_n$-OH, n is DP):

Red: $[M+H]^+$, DP = 7-12 from left to right

Blue: $[M+Na]^+$, DP = 7-12 from left to right

Green: $[M+K]^+$, DP = 7-12 from left to right

Orange: $[M-C_6H_5CH_2OH+Na]^+$, DP = 8-12 from left to right

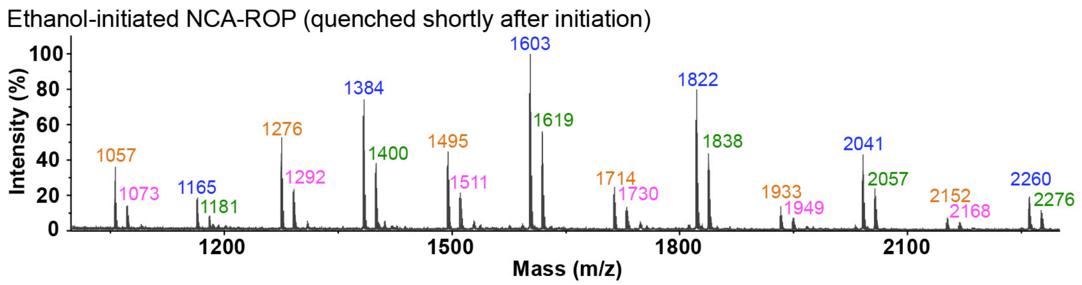

Supplementary Fig. 8. MALDI-TOF MS spectrum of PBLG with low molecular weight from ethanol-initiated NCA-ROP on UiO-66 NPs. UiO-66 NPs were washed with ethanol and dried before use. The polymerization was quenched shortly after initiation to control the molecular weight so that MALDI analysis can be facilitated.

Peak assignments (M = H-(NH-($C_{11}H_{12}O_2$)-CO)$_n$-OCH_2CH_3, n is DP):

Blue: $[M+Na]^+$, DP = 5-10 from left to right

Green: $[M+K]^+$, DP = 5-10 from left to right

Orange: $[M-C_6H_5CH_2OH+Na]^+$, DP = 5-10 from left to right

Purple: $[M-C_6H_5CH_2OH+K]^+$, DP = 5-10 from left to right

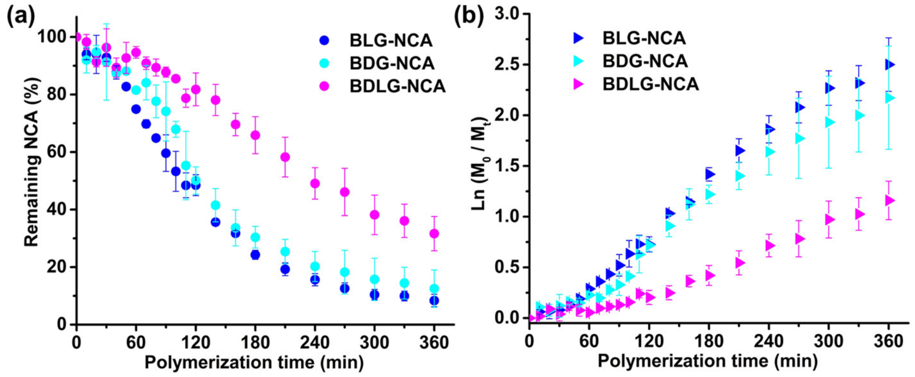

Supplementary Fig. 9. Kinetic study on UiO-66-1 nanoparticle-mediated ROP of BLG-NCA, BDG-NCA or BDLG-NCA by FT-IR. (a) NCA monomer conversion versus time. (b) $\ln(M_0/M_t)$ versus time. Condition: $[NCA]_0$ = 0.114 M, m_{NCA}/m_{UiO-66} = 10, anhydrous DCM was used. Error bars represent ±SD.

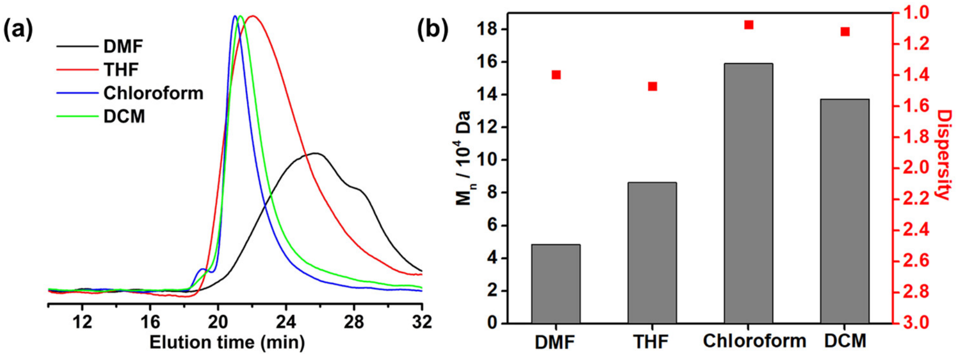

Supplementary Fig. 10. GPC characterization results of PBLGs prepared using UiO-66-3 NP-mediated NCA-ROPs in different anhydrous solvents. Reaction time: 20 h, $m_{BLG-NCA}/m_{UiO-66}$ = 10. (a) dRI curve overlay of the obtained PBLGs. (b) Comparison on M_n and dispersity of the obtained PBLGs.

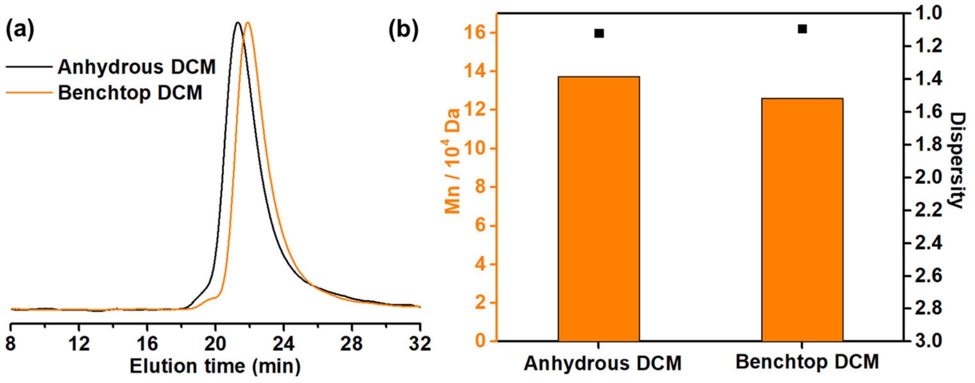

Supplementary Fig. 11. GPC characterization results of PBLGs prepared using UiO-66-3 NP-mediated NCA-ROPs in anhydrous DCM or benchtop DCM (not dried). Reaction time: 12 h, $m_{NCA}/m_{UiO\text{-}66}$ = 10. (a) dRI curve overlay of the obtained PBLGs. (b) Comparison on M_n and dispersity of the obtained PBLGs.

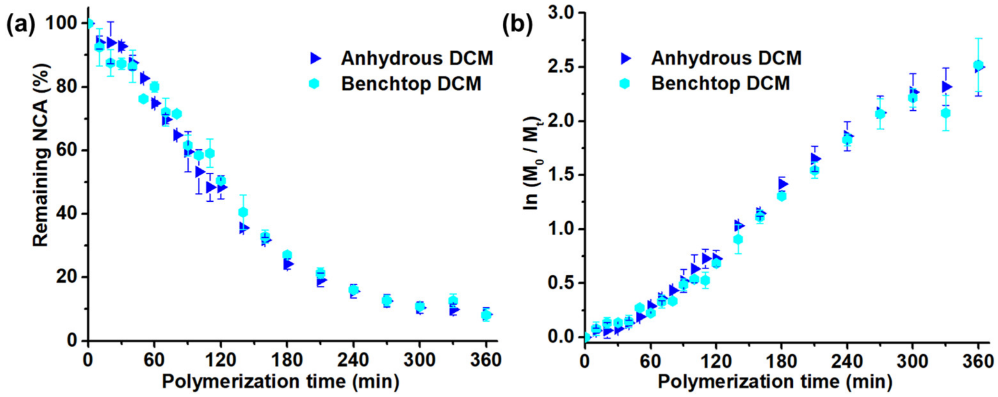

Supplementary Fig. 12. Kinetic study on UiO-66-1 nanoparticle-mediated ROP of BLG-NCA in anhydrous DCM or benchtop DCM (not dried) by FT-IR. Condition: $[BLG\text{-}NCA]_0$ = 0.114 M and $m_{BLG\text{-}CA}/m_{UiO\text{-}66}$=10. (a) NCA monomer conversion versus time. (b) $\ln(M_0/M_t)$ versus time. Error bars represent ±SD.

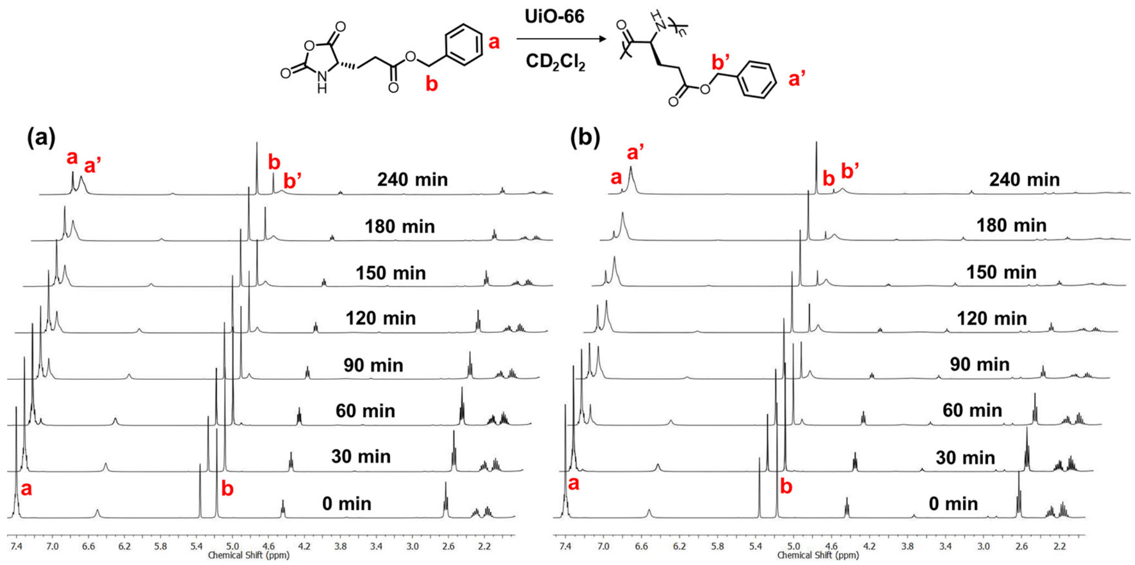

Supplementary Fig. 13. The monitoring of UiO-66-1 NP-mediated BLG-NCA polymerization by *in situ* ^{1}H NMR ([BLG-NCA]$_0$ = 0.114 M, in CD_2Cl_2). (a) Spectra overlay for NCA-ROP with a feeding mass ratio $m_{\text{BLG-NCA}}/m_{\text{UiO-66}}$ = 10; (b) Spectra overlay for NCA-ROP with a feeding mass ratio $m_{\text{BLG-NCA}}/m_{\text{UiO-66}}$ = 5.

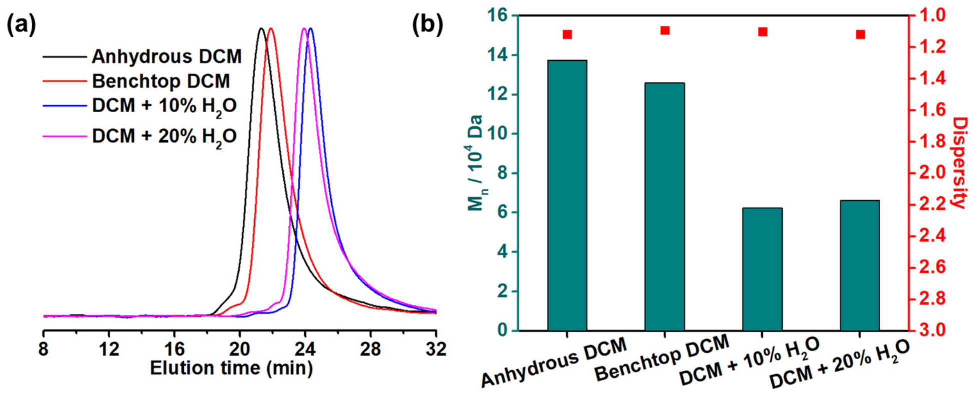

Supplementary Fig. 14. GPC characterization results of PBLGs prepared using UiO-66-3 NP-mediated NCA-ROPs in anhydrous DCM, benchtop DCM (not dried) or DCM-water biphasic mixture. Reaction time: 12 h, $m_{\text{BLG-NCA}}/m_{\text{UiO-66}}$ = 10. (a) dRI curve overlay of the obtained PBLGs. (b) Comparison on M_n and dispersity of the obtained PBLGs.

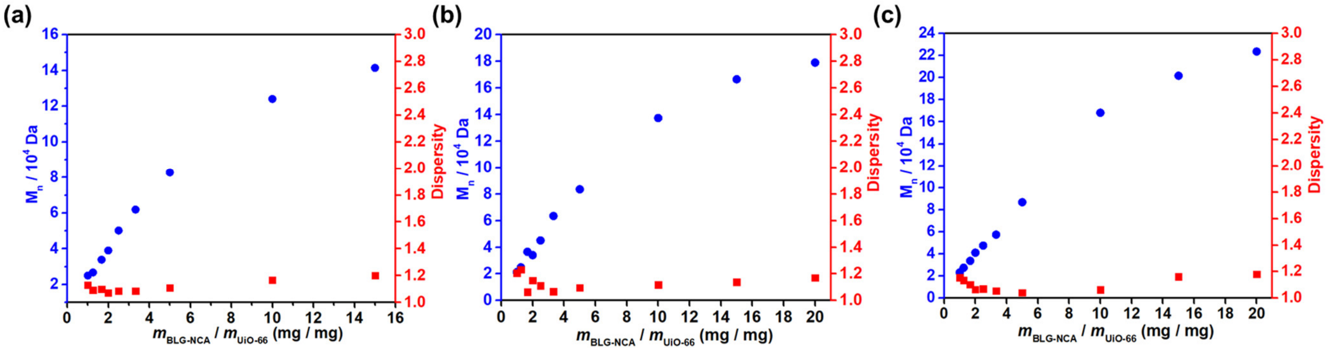

Supplementary Fig. 15. M_n and dispersity of the PBLGs obtained from the water-initiated NCA-ROPs on UiO-66 nanoparticles at different $m_{BLG\text{-}NCA}/m_{UiO\text{-}66}$ ratios. (a) Polymerizations done using UiO-66-1, 60~80 nm. (b) Polymerizations done using UiO-66-3, 250~300 nm. (c) Polymerizations done using UiO-66-4, 550~600 nm.

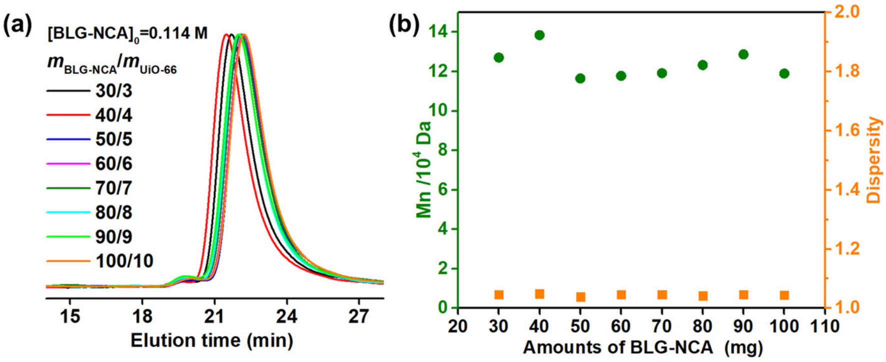

Supplementary Fig. 16. Predictability and reproducibility of M_n and dispersity in NCA-ROPs mediated by UiO-66-2. Conditions: $m_{BLG\text{-}NCA}/m_{UiO\text{-}66}$ = 10, [BLG-NCA]$_0$ = 0.114 M, anhydrous DCM of varied volumes. (a) Overlay of GPC-dRI curves of the obtained PBLGs, ratios are shown in mg/mg; (b) Comparison on M_n and dispersity of the obtained PBLGs.

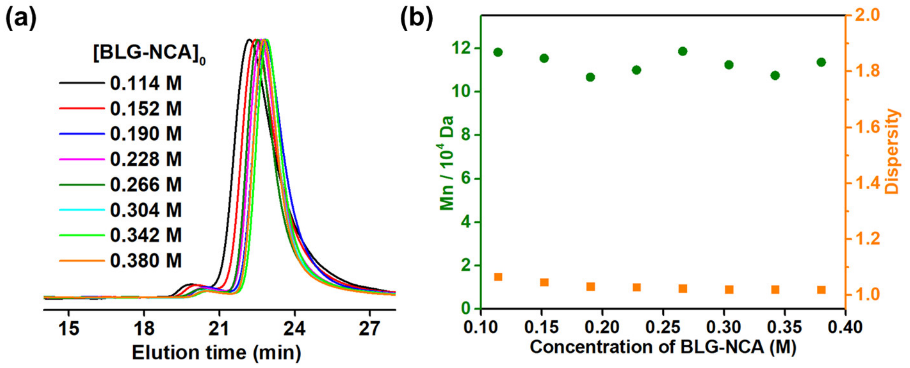

Supplementary Fig. 17. Concentration effects on M_n and dispersity of PBLGs obtained from NCA-ROPs mediated by UiO-66-2. Condition: $m_{BLG\text{-}NCA}/m_{UiO\text{-}66}$ = 10, 1 mL of anhydrous DCM: (a) Overlay of GPC-dRI curves of the obtained PBLGs; (b) Comparison on the M_n and dispersity of the obtained PBLGs.

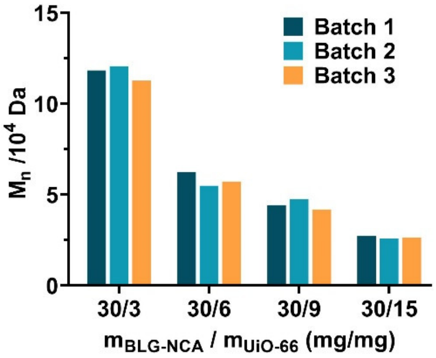

Supplementary Fig. 18. The molecular weights of PBLG synthesized by different batches of UiO-66 nanoparticles using different BLG-NCA/MOF mass ratios. For all the products, the measured dispersity was < 1.1.

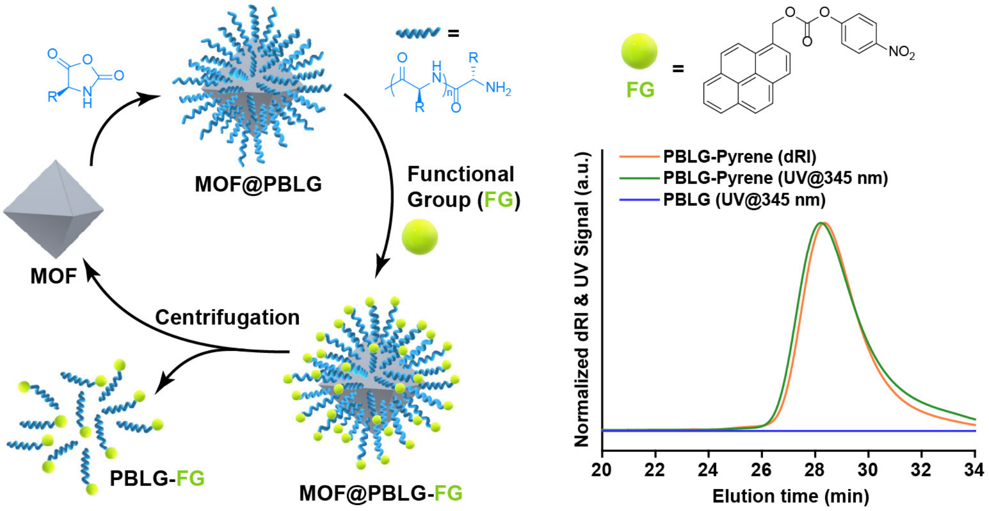

Supplementary Fig. 19. Left: a schematic illustration of the polypeptides' end-group modification process using the MOF-catalyzed NCA polymerization strategy. Right: a GPC curve overlay verifying the successful end-group modification of PBLG from the MOF-catalyzed NCA polymerization, using the amine-reactive pyrenemethyl nitrophenyl carbonate.

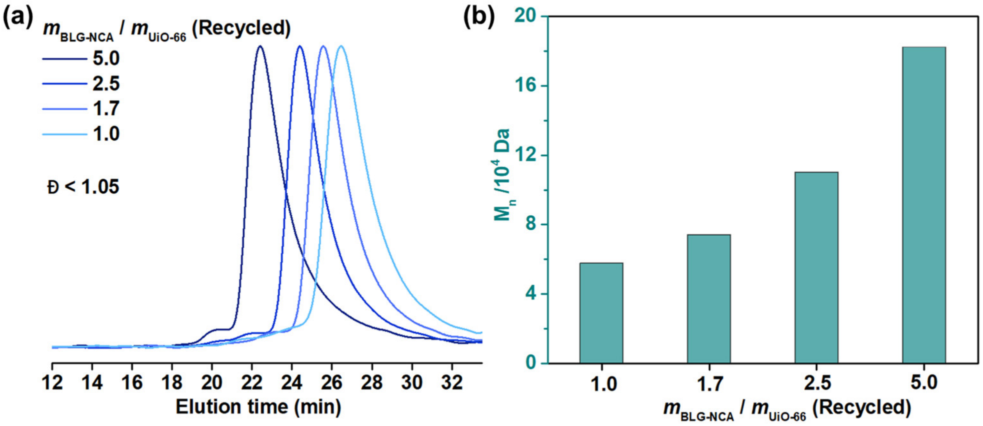

Supplementary Fig. 20. The well-controlled polymerization of BLG-NCA by the recycled UiO-66 nanoparticles with varied mass ratio of BLG-NCA to the UiO-66 nanoparticles. $[BLG\text{-}NCA]_0 = 0.114$ M, anhydrous DCM as solvent. (a) The GPC-dRI curves of the obtained polypeptides. (b) Comparison on M_n of the obtained PBLGs.

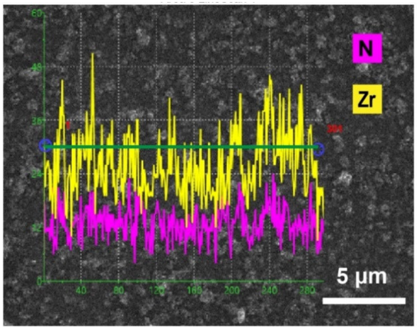

Supplementary Fig. 21. Element density profile of the UiO-66-PBLG hybrid material obtained from EDX line-scan ($m_{BLG\text{-}NCA}/m_{UiO\text{-}66}$ = 2 in the preparation, 37.5 wt.% UiO-66-1 in the material).

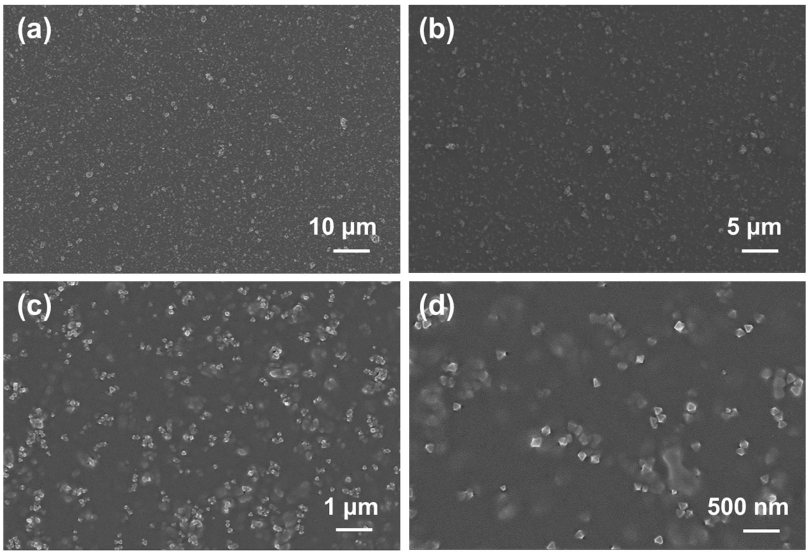

Supplementary Fig. 22. SEM images of the UiO-66@PBLG hybrid material prepared using the PBLG-on-UiO-66 complex directly from the ROP of BLG-NCA mediated by UiO-66 NPs ($m_{BLG\text{-}NCA}/m_{UiO\text{-}66}$ = 10). The membrane contains 10 wt.% UiO-66-1 nanoparticles. Images were taken at (a) 1.00 kx magnification; (b) 2.00 kx magnification; (c) 10.00 kx magnification; (d) 20.00 kx magnification.

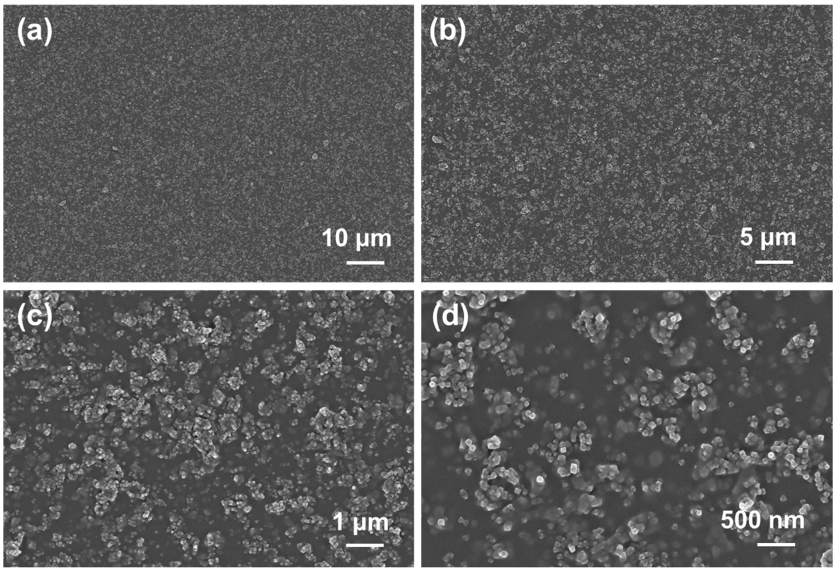

Supplementary Fig. 23. SEM images of the UiO-66@PBLG hybrid material prepared using the PBLG-on-UiO-66 complex directly from the ROP of BLG-NCA mediated by UiO-66 NPs ($m_{\text{BLG-NCA}}/m_{\text{UiO-66}} = 6$). The membrane contains 19 wt.% UiO-66-1 nanoparticles. Images were taken at (a) 1.00 kx magnification; (b) 2.00 kx magnification; (c) 10.00 kx magnification; (d) 20.00 kx magnification.

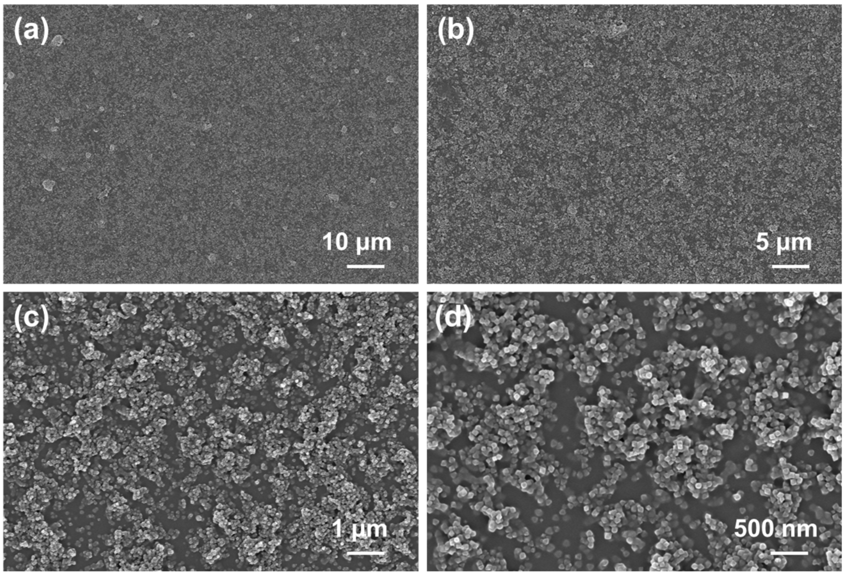

Supplementary Fig. 24. Top-view SEM images of the UiO-66@PBLG hybrid material prepared using the PBLG-on-UiO-66 complex directly from the ROP of BLG-NCA mediated by UiO-66 NPs ($m_{\text{BLG-NCA}}/m_{\text{UiO-66}} = 2$). The membrane contains 38.5 wt.% UiO-66-1 nanoparticles. Images were taken at (a) 1.00 kx magnification; (b) 2.00 kx magnification; (c) 10.00 kx magnification; (d) 20.00 kx magnification.

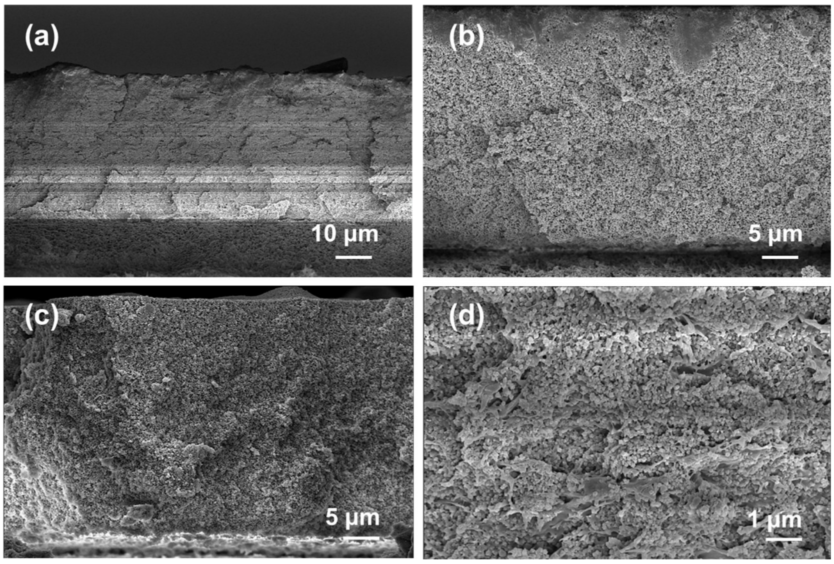

Supplementary Fig. 25. Side-view SEM section images of the UiO-66@PBLG MMM prepared using the PBLG-on-UiO-66 complex directly from the ROP of BLG-NCA mediated by UiO-66 NPs ($m_{\text{BLG-NCA}}/m_{\text{UiO-66}} = 2$). The MMM contains 38.5 wt.% UiO-66-1 nanoparticles. Images were taken at (a) 1.00 kx magnification; (b-c) 2.00 kx magnification; (d) 10.00 kx magnification.

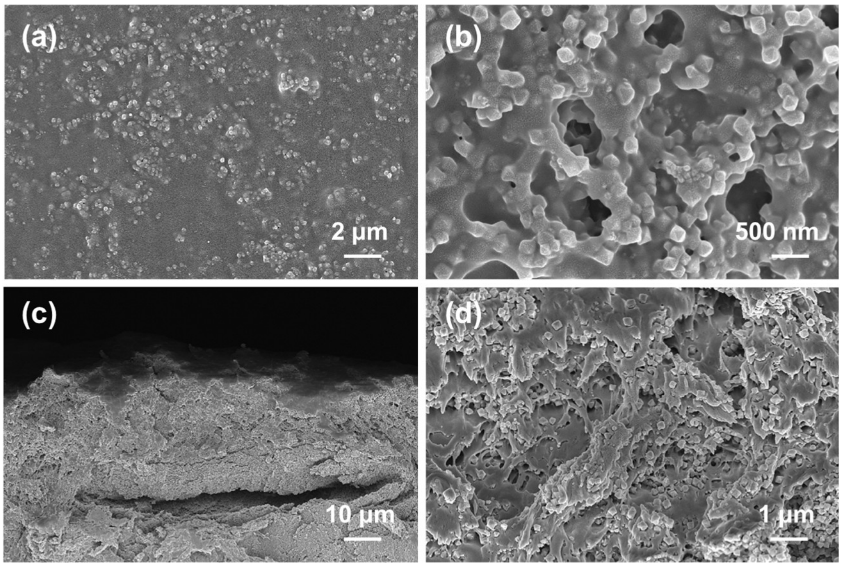

Supplementary Fig. 26. SEM images of the UiO-66+PBLG MMM prepared by physically mixing UiO-66 NPs with PBLG, the MMM contains 38.5 wt.% UiO-66-1 nanoparticles. (a) Surface morphology of the UiO-66+PBLG MMM at 5.00 kx magnification. (b) Surface morphology of the UiO-66+PBLG MMM at 20.00 kx magnification. (c) Section morphology of the UiO-66+PBLG MMM at 1.00 kx magnification. (d) Section morphology of the UiO-66+PBLG MMM at 10.00 kx magnification.

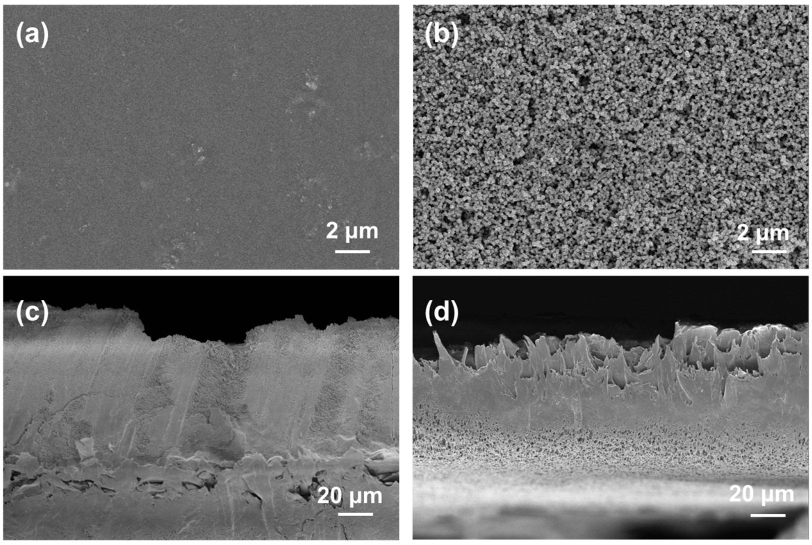

Supplementary Fig. 27. SEM images of the PBLG membrane (a, c) and the UiO-66 Nylon membrane (b, d). (a-b) Surface morphology, (c-d) section morphology.

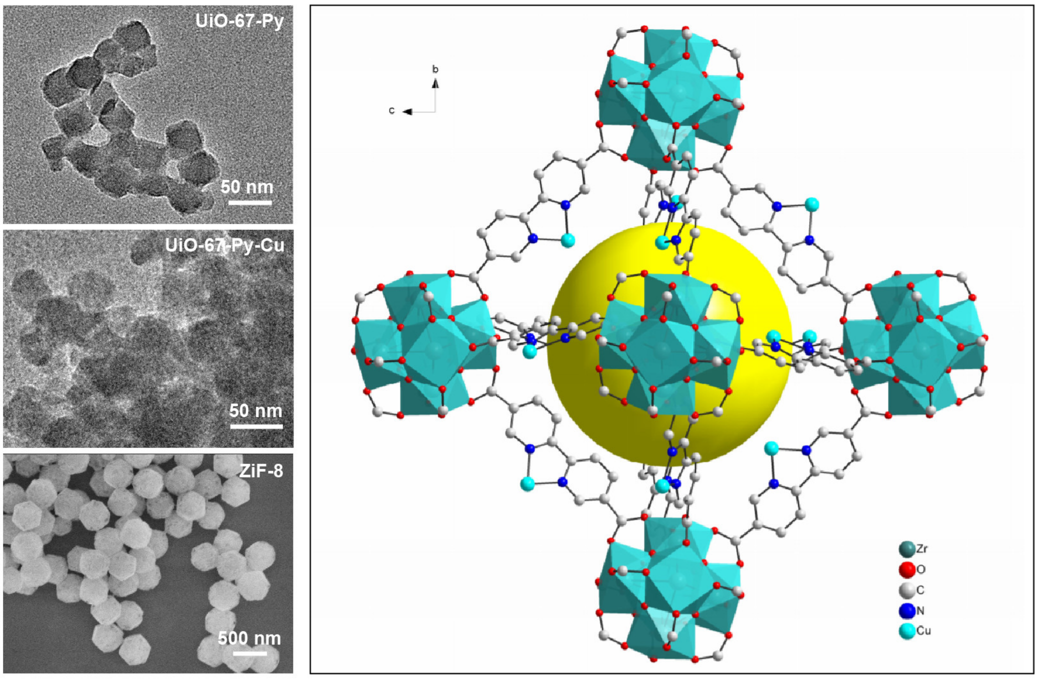

Supplementary Fig. 28. Left: TEM images of UiO-67-Py, UiO-67-Py-Cu and ZiF-8 used for MMM materials preparation. Right: Structure illustration of UiO-67-Py-Cu.

Table S1. ICP-MS quantification of leaked Zr species in different polymerization conditions.

Solvent	Anhydrous DCM	10% H_2O/DCM mixture
Zr content (ppb)	0.19	0.25

Table S2. Calculated pseudo-first-order rate constants of NCA-ROPs with different feeding mass ratios of BLG-NCA to UiO-66 nanoparticles. The rate constant of TMS[a] amine-initiated NCA-ROP was provided for comparison.

Entry	$m_{\text{BLG-NCA}}/m_{\text{UiO-66}}$ (mg/mg)	UiO-66 Conc. (mg/mL)	k (min^{-1})	*Pearson*'s *R*
1	10	3	0.0096	0.9984
2	5	6	0.0184	0.9986
3	2.5	12	0.0321	0.9937

[a] Trimethylsilyl (TMS) amine initiated BLG polymerization in DCM: k = 0.0054 min^{-1}

Table S3. The cost of synthesized MOF and other initiators were displayed as below:

	MOF NPs[a]	Macroinitiator PNB_n (Ref 11)	"SIMPLE" (Ref 15)	PAMAM G2-G5 (Ref 18)	TBAA (Ref 19)
Cost (USD/g)	3.6	189	51.9	547-2623	22.2

[a] Considering recycled uses for 3 times.

Table S4. ICP-MS quantification of Zr and Cu contents in UiO-67-Py-Cu.

Entry	Zr	Cu
Measured Concentration[a] (mg/L)	14.92	9.24
Atomic Mass (g/mol)	91.224	63.546
Molar Ratio (Zr as 1)	1	0.889

[a] Protocol: 8.51 mg of UiO-67-Py-Cu was dissolved in 2 mL of 40% HF, and 18 mL of 5% HNO_3 was added to obtain the digestion solution. After full digestion, 16 mL of 5% HNO_3 was added to 4 mL of the digestion solution to obtain the solution for ICP measurements.

NMR characterizations

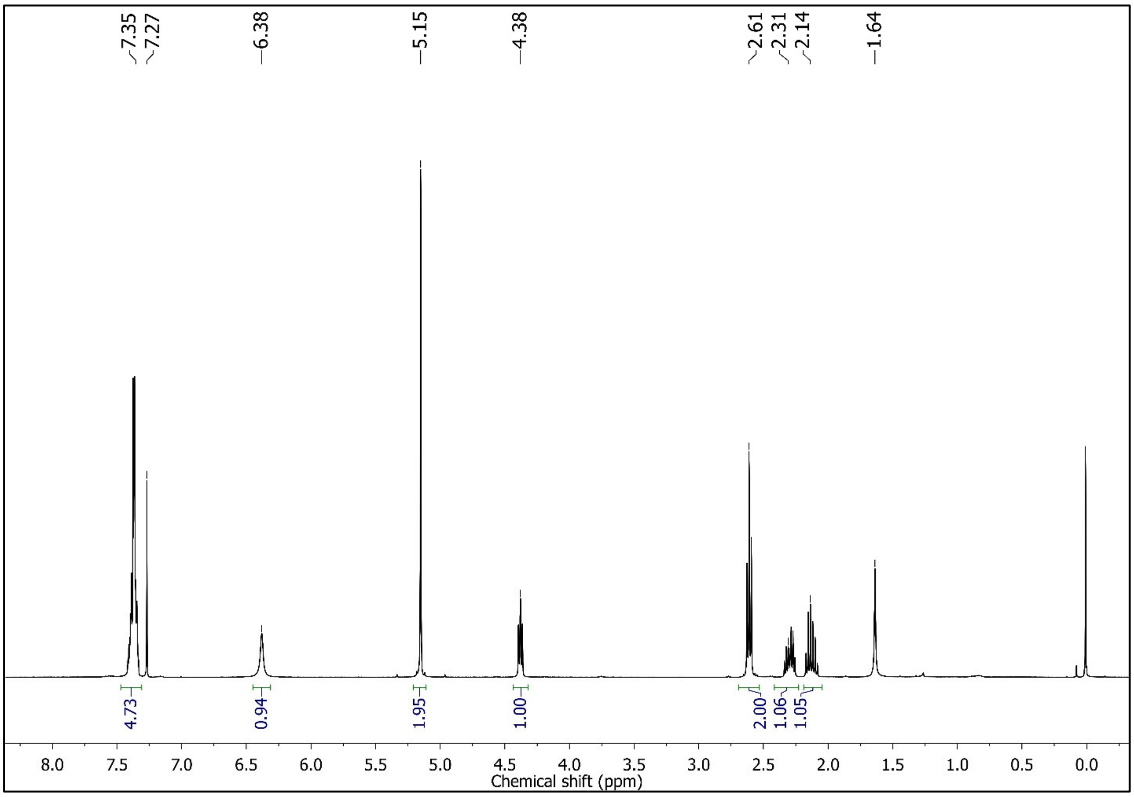

Supplementary Fig. 29. ^{1}H NMR spectrum of BLG-NCA monomer in $CDCl_3$.

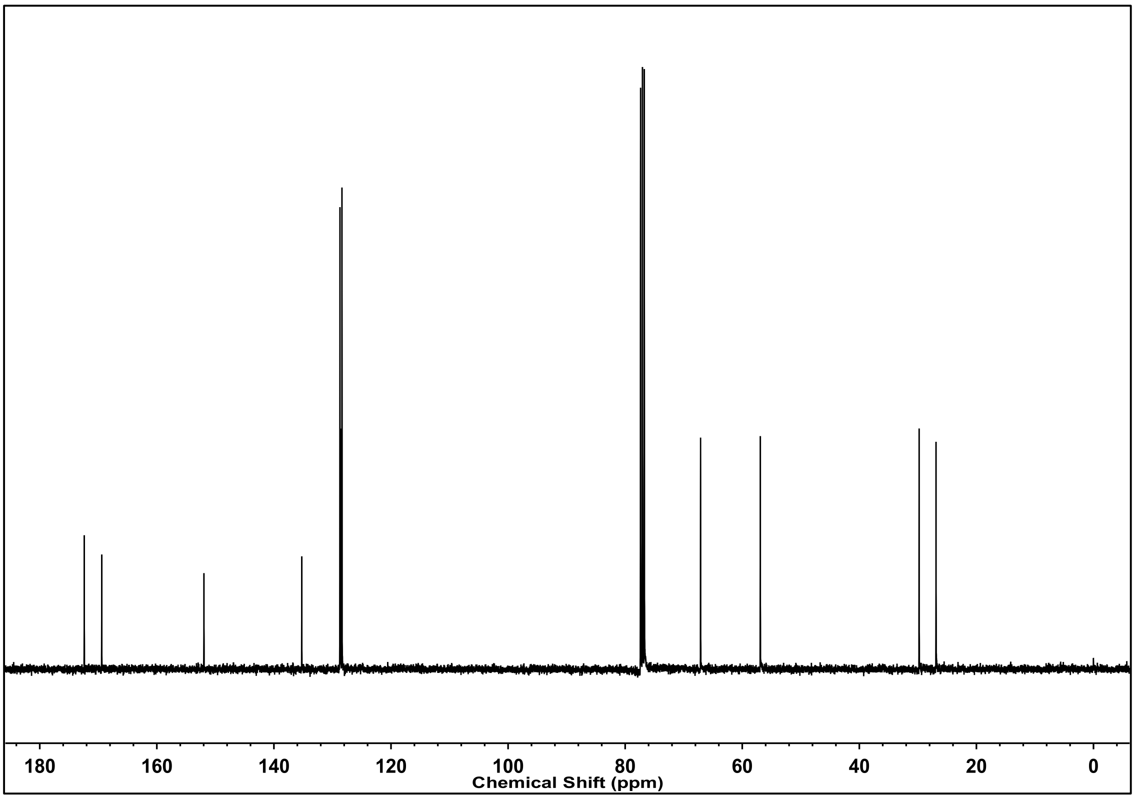

Supplementary Fig. 30. ^{13}C NMR spectrum of BLG-NCA monomer in $CDCl_3$.

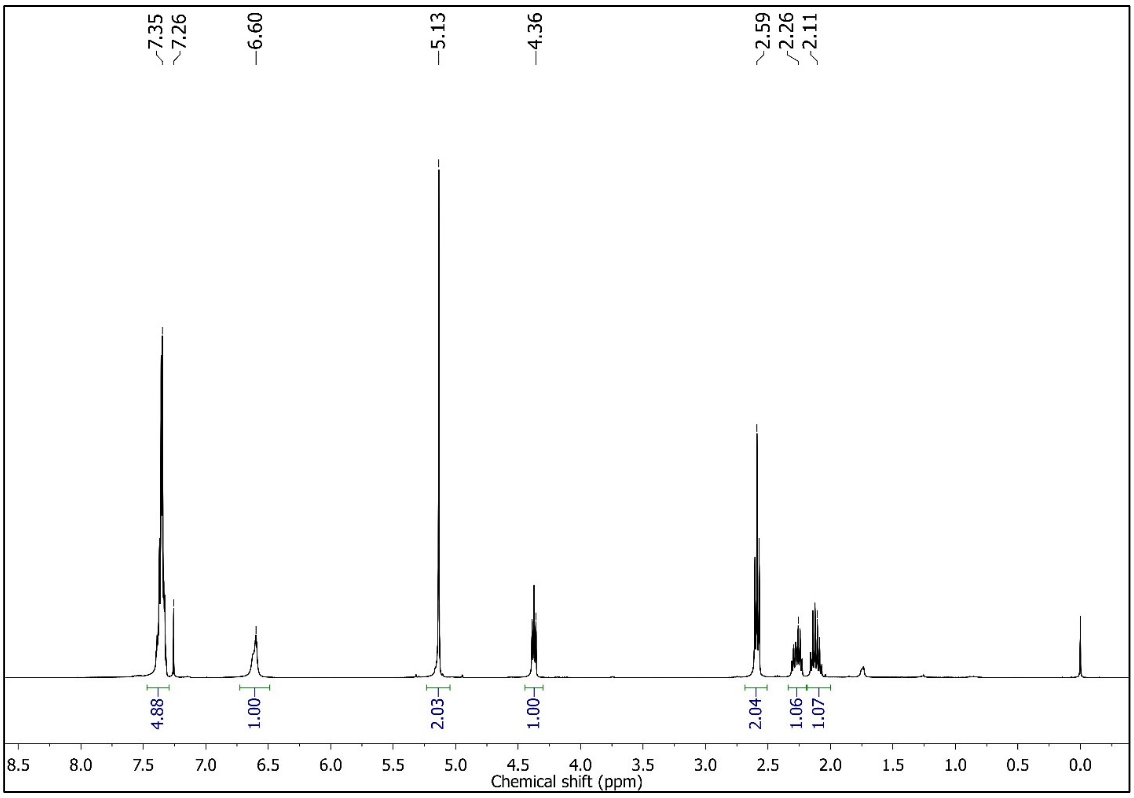

Supplementary Fig. 31. ^{1}H NMR spectrum of BDG-NCA monomer in $CDCl_3$.

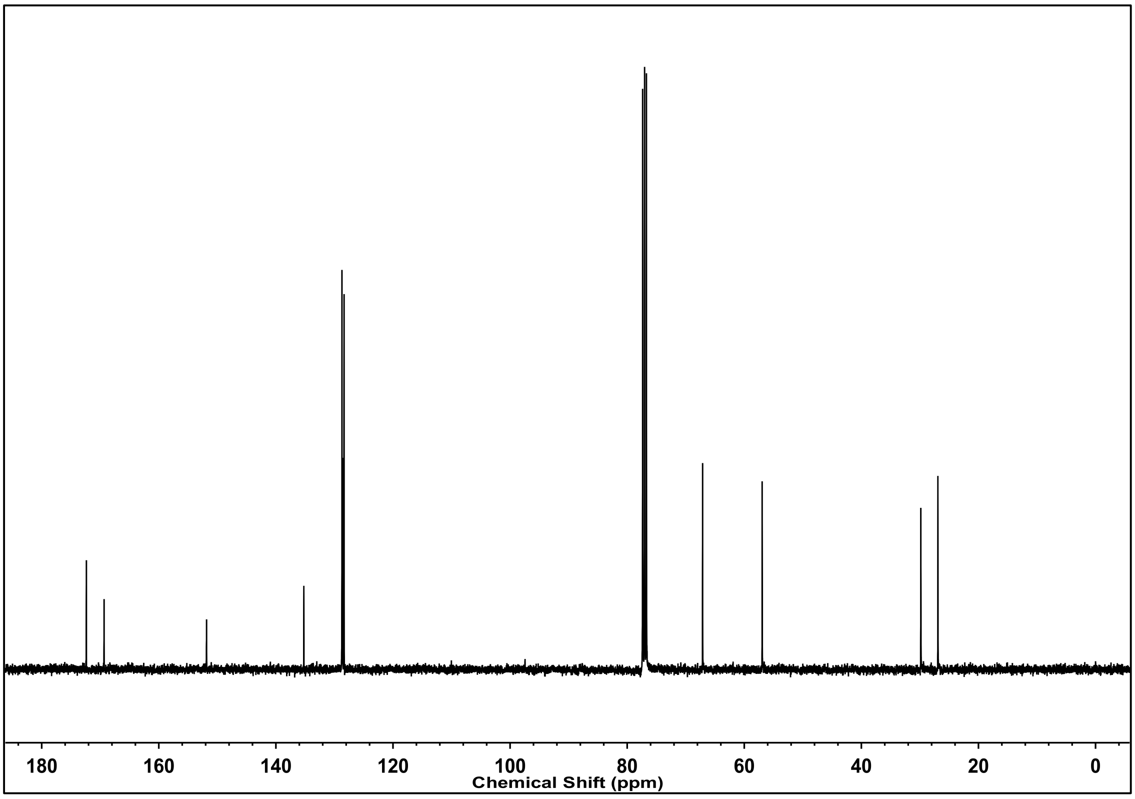

Supplementary Fig. 32. ^{13}C NMR spectrum of BDG-NCA monomer in $CDCl_3$.

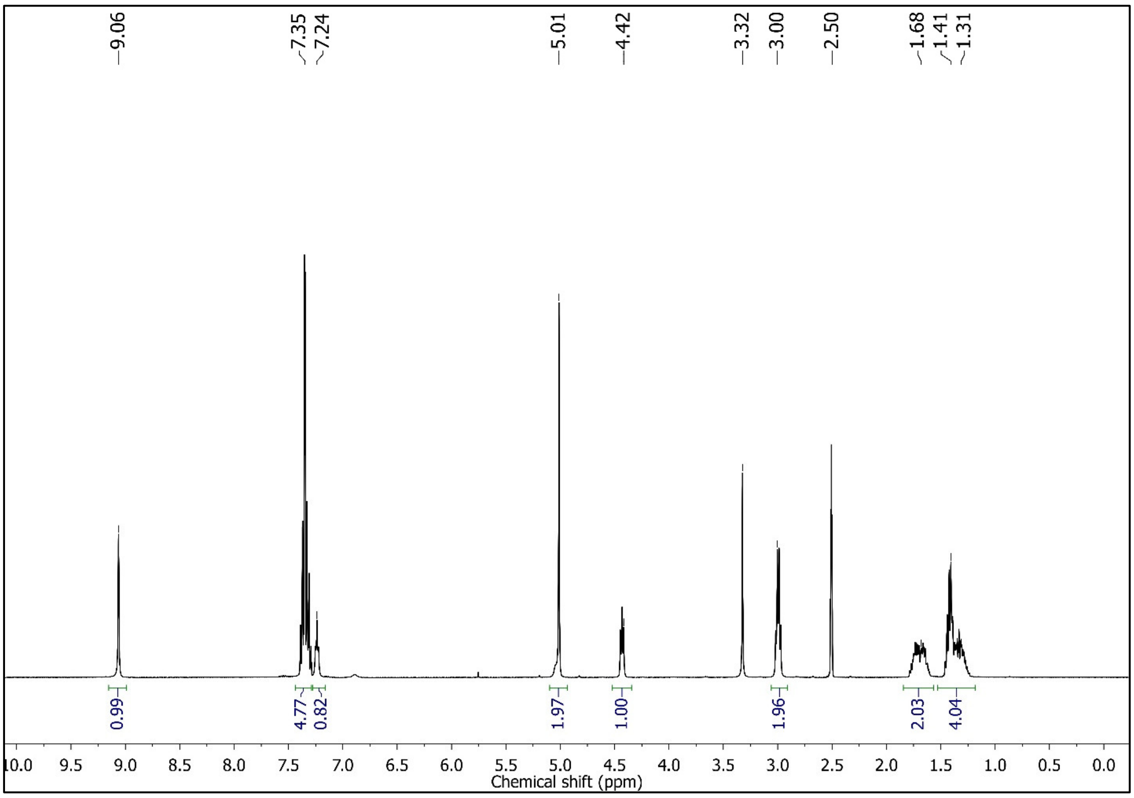

Supplementary Fig. 33. ^{1}H NMR spectrum of ZLL-NCA monomer in DMSO-d_6.

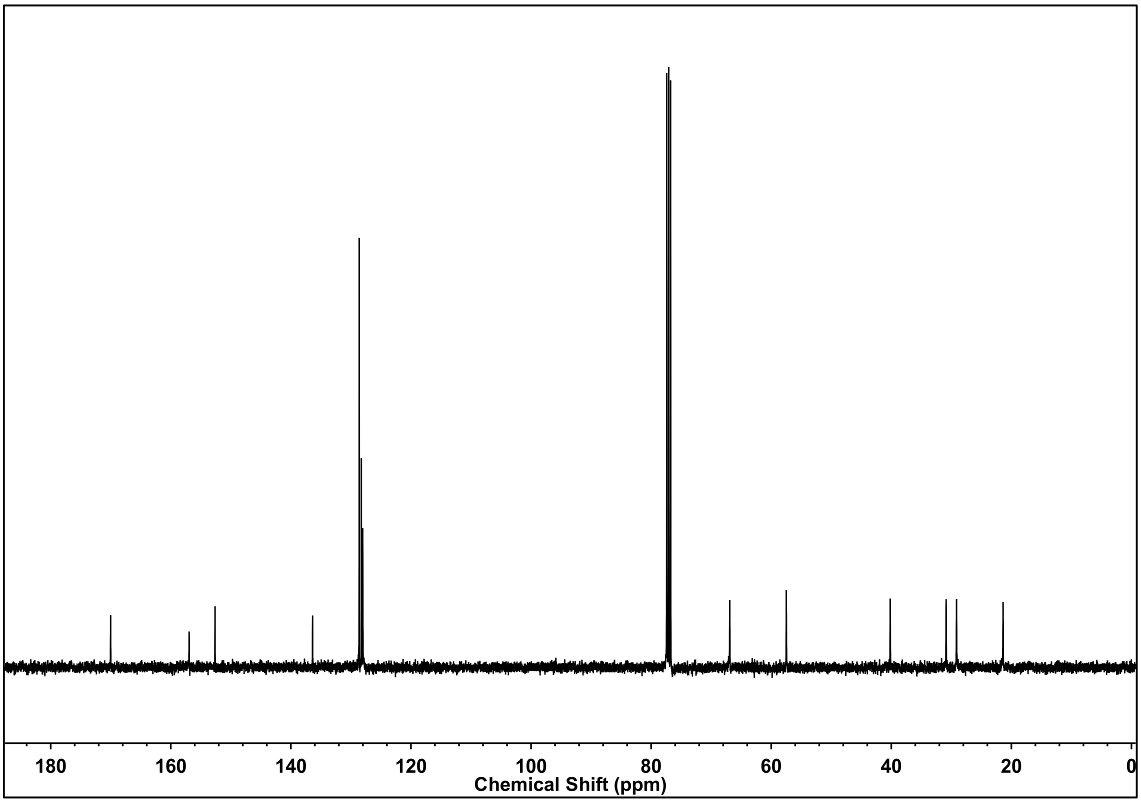

Supplementary Fig. 34. ^{13}C NMR spectrum of ZLL-NCA monomer in $CDCl_3$.

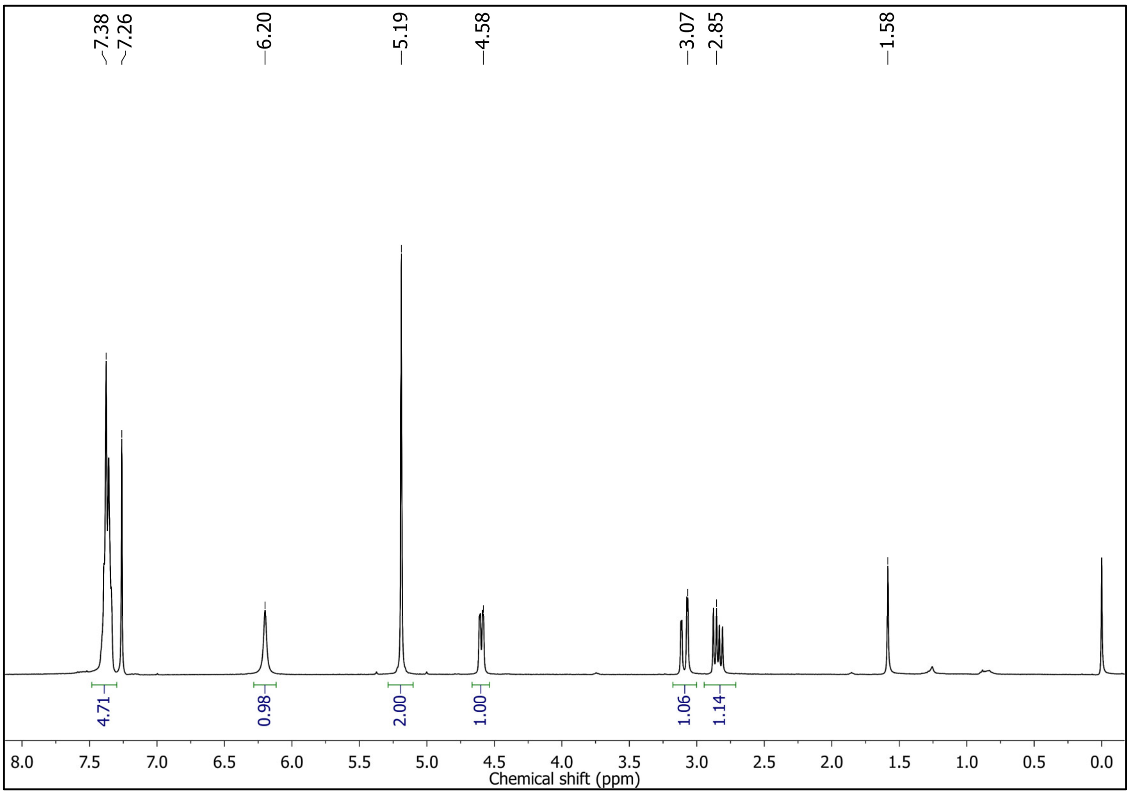

Supplementary Fig. 35. ^{1}H NMR spectrum of BLA-NCA monomer in $CDCl_3$.

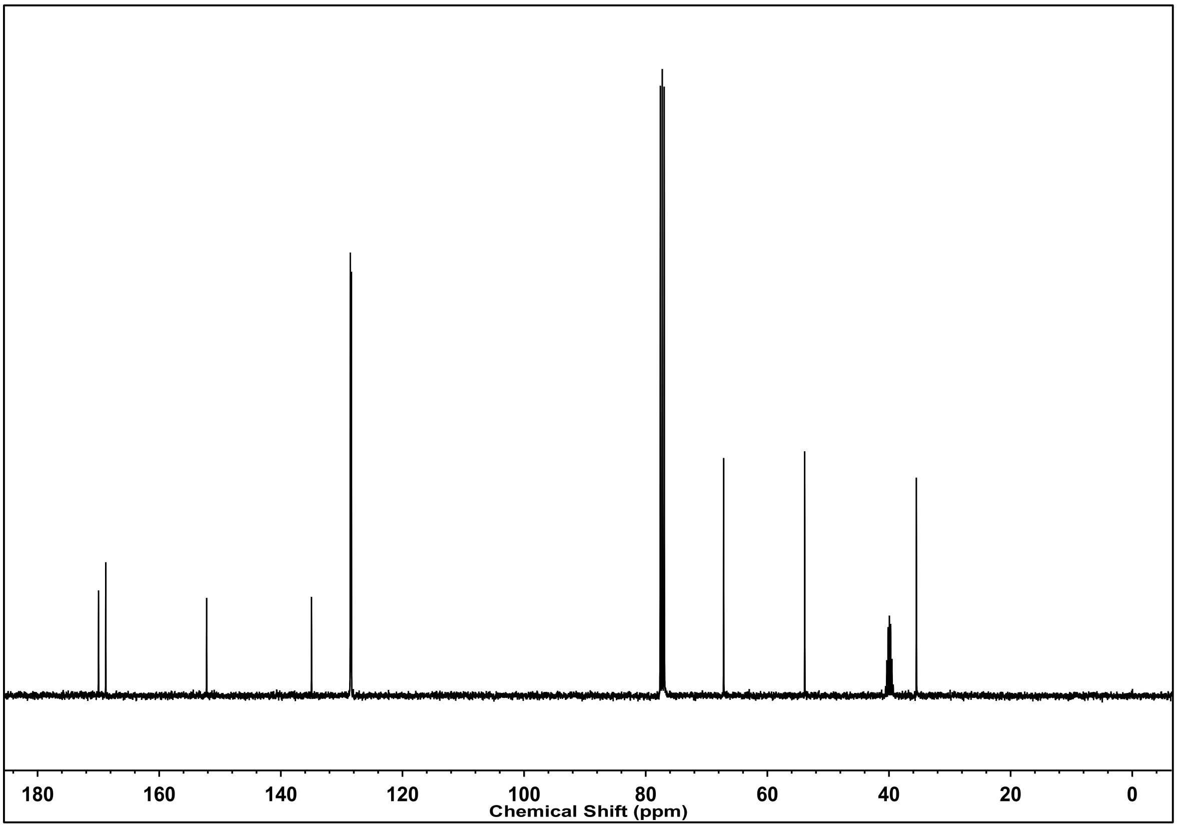

Supplementary Fig. 36. ^{13}C NMR spectrum of BLA-NCA monomer in $CDCl_3$.

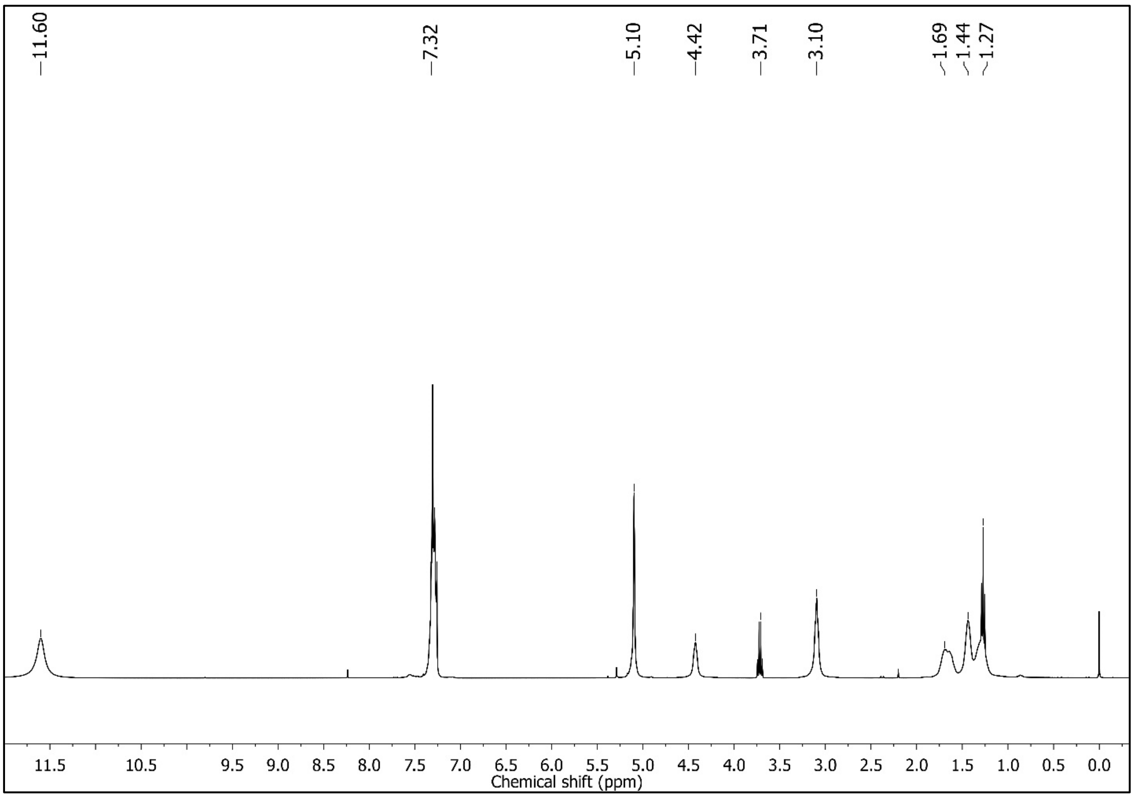

Supplementary Fig. 37. ^1H NMR spectrum of PZLL obtained using UiO-66 NP-mediated NCA-ROP strategy. 15% TFA-*d* in $CDCl_3$ was used as the solvent.

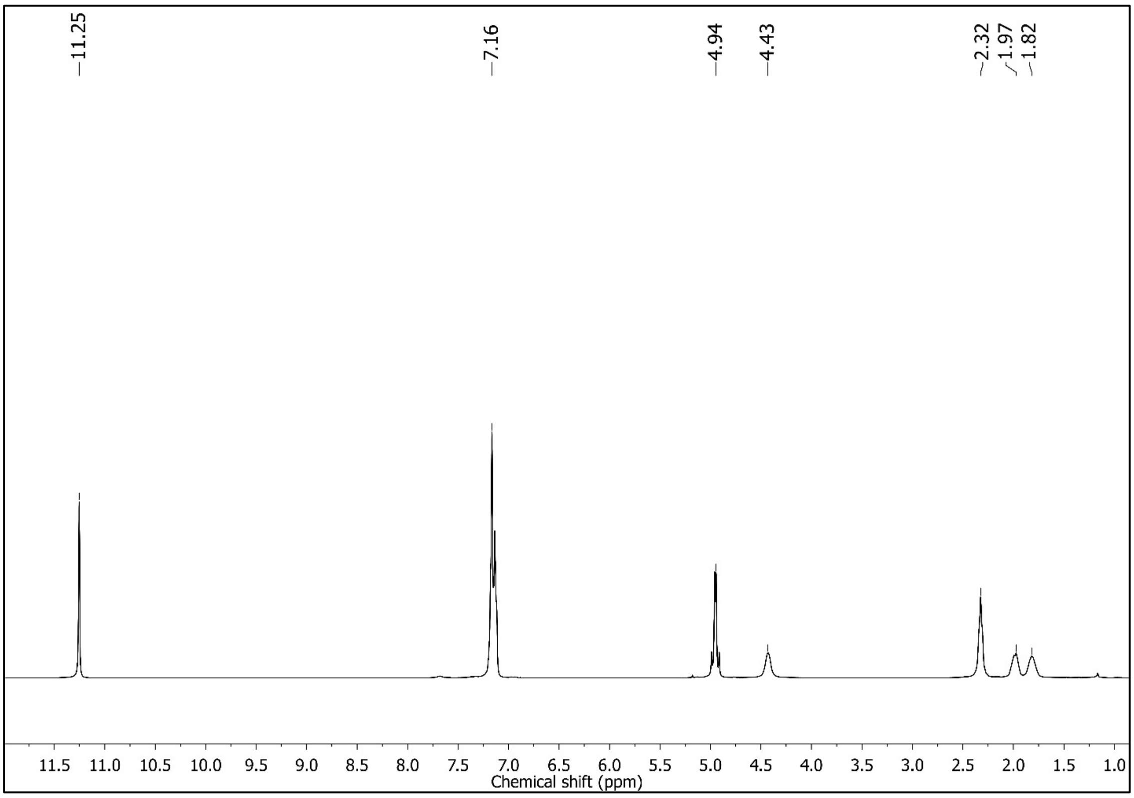

Supplementary Fig. 38. ^1H NMR spectrum of PBLG obtained using UiO-66 NP-mediated NCA-ROP strategy. 15% TFA-*d* in $CDCl_3$ was used as the solvent.

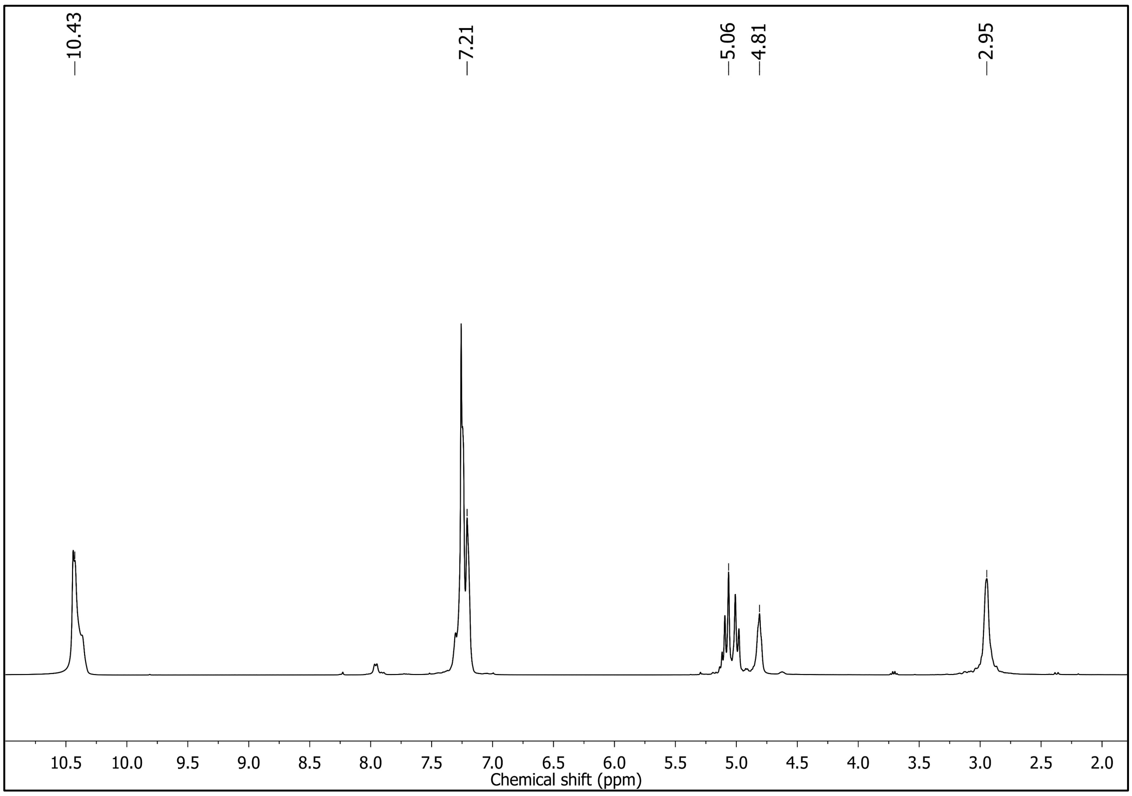

Supplementary Fig. 39. ^{1}H NMR spectrum of PBLA obtained using UiO-66 NP-mediated NCA-ROP strategy. 15% TFA-*d* in $CDCl_3$ was used as the solvent.

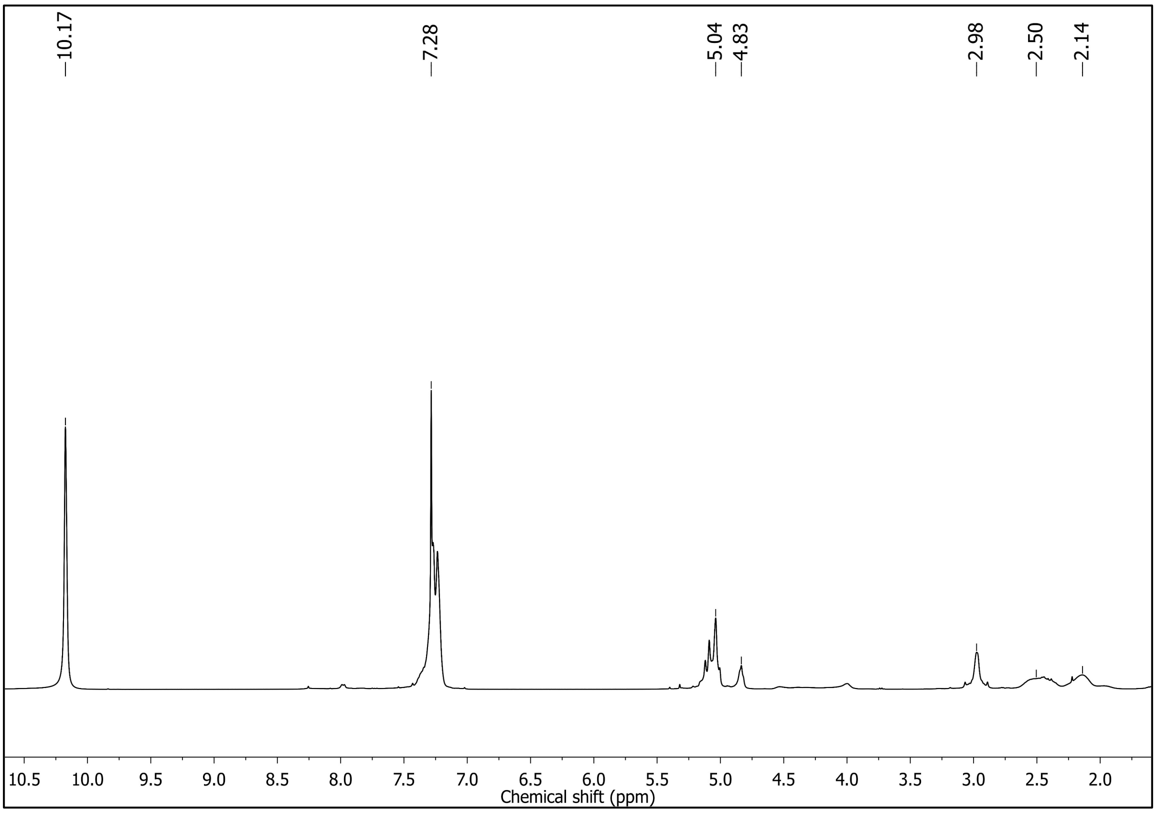

Supplementary Fig. 40. ^{1}H NMR spectrum of PBLA-PBLG copolymer obtained using UiO-66 NP-mediated NCA-ROP strategy. 15% TFA-*d* in $CDCl_3$ was used as the solvent.

Additional discussions

Discussion 1. Effect of filtration on MOF@PBLG.

The effect of filtration on MOF@PBLG varies based on the size of MOF used in the preparation. For very small MOF particles, the resulting MOF@PBLG can pass through most filter membranes. If large MOF particles (550-600 µm in diameter) are used for the preparation, common syringe filters (0.22 or 0.45 µm ones) can be used for filtration. The filtration process itself (requiring decent pressure) applies a strong shear force to the structures on nanoparticles, and similar to centrifugation, such force can lead to the detachment of the polypeptides. This process is like the detachment of conjugated proteins on gold nanoparticles during filtration.

Experimentally, by forcefully passing the MOF@PBLG mixture through a 0.22 µm PTFE filter, approximately 20% polypeptide was found detached from the MOF. Considering the known sensitivity of this MOF@PBLG material to mechanical force (centrifugation), and the excellent polymerization control with an accelerated polymerization mechanism (detached polymer chains would not enjoy acceleration thus will harm the overall dispersity control, but this was not observed), we tentatively believe that the polymer chains mostly remain attached to the MOF surface during the polymerization process, i.e., the polypeptides detected in the filtrate were caused by the filtration process.

Discussion 2. Optimization of polymerization protocol.

Shoulder peaks may appear sometimes in the GPC-dRI curves was due to the unsynchronous initiation of the NCA-ROP on the MOF nanoparticles' surfaces. This was likely resulted from the heterogeneous nature of this initiation system, and may be related to stirring speed, concentration, and other factors. We found that the shoulder peaks were more likely to appear when the stirring was not consistent. For example, when several reaction vials were bundled together with a rubber band and placed on a stirring plate together for the polymerization, shoulder peaks were likely to appear. The stirring bars in different vials might have affected each other, or they can be far away from the stirring plate's center, leading to inconsistent stirring that reduces reaction mixture homogeneity, thus the emergence of the shoulder peaks. From our tests, we believe that a stirring rate of 800 is good enough, and individual vials are better placed on different mini stirring plates for the best results. This simple optimization to the protocol can largely remove the shoulder peaks in the GPC-dRI curves for polymerizations. However, at high NCA/MOF ratios, weak should peaks may still be observed (see Supplementary Fig. 41, left) and they were likely caused by catalyst heterogeneity that was not resolved by stirring. The reason we speculated was the partial aggregation of the MOF particles. When NCA/MOF ratio was high, NCAs competed more for the reactive sites on the MOF, thus the effect of aggregation was magnified. To address this issue, MOF NPs could be pre-dispersed in the polymerization solvent by applying ultrasonic treatment for 40 minutes before monomer addition. This pre-dispersion technique again improved the shape of the GPC peaks, leading to diminished shoulder peaks (Supplementary Fig. 41, right).

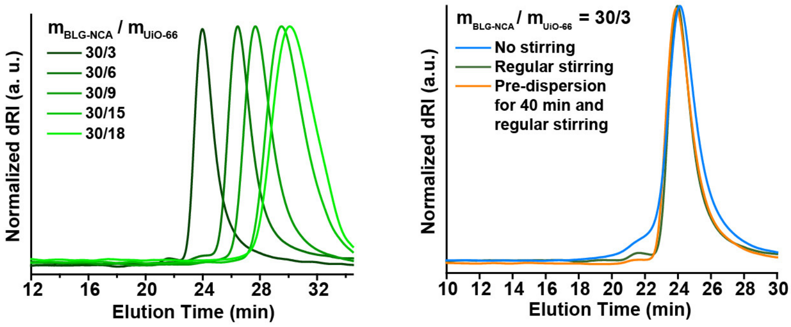

Supplementary Fig. 41. The effect of stirring and pre-dispersion (ultrasonication) on the NCA-ROP.

Supplementary References

1. Baumgartner, R.; Fu, H.; Song, Z.; Lin, Y.; Cheng, J., Cooperative polymerization of alpha-helices induced by macromolecular architecture. *Nat. Chem.* **2017,** *9* (7), 614-622.
2. Pan, Y.; Liu, Y.; Zeng, G.; Zhao, L.; Lai, Z., Rapid synthesis of zeolitic imidazolate framework-8 (ZIF-8) nanocrystals in an aqueous system. *Chem. Commun.* **2011,** *47* (7), 2071-3.
3. Umemura, A.; Diring, S.; Furukawa, S.; Uehara, H.; Tsuruoka, T.; Kitagawa, S., Morphology Design of Porous Coordination Polymer Crystals by Coordination Modulation. *J. Am. Chem. Soc.* **2011,** *133* (39), 15506-15513.
4. Schaate, A.; Roy, P.; Godt, A.; Lippke, J.; Waltz, F.; Wiebcke, M.; Behrens, P., Modulated synthesis of Zr-based metal-organic frameworks: from nano to single crystals. *Chemistry* **2011,** *17* (24), 6643-51.
5. Wang, T. C.; Vermeulen, N. A.; Kim, I. S.; Martinson, A. B.; Stoddart, J. F.; Hupp, J. T.; Farha, O. K., Scalable synthesis and post-modification of a mesoporous metal-organic framework called NU-1000. *Nat. Protoc.* **2016,** *11* (1), 149-62.
